# Supplementary material for: Incidence of vasa praevia: a systematic review and meta-analysis
Source: BMJ Open. 2023 Sep 20;13(9):e075245. doi: 10.1136/bmjopen-2023-075245 (PMC10514663; doi:10.1136/bmjopen-2023-075245)
Supplement: Supplementary data [file bmjopen-2023-075245supp002.pdf]

Supplementary table S1: Search Strategy for study

**MEDLINE**
1. Medline(Vasa praevia OR vasa previa).ti,ab<br>2. Medline(abnormal cord insertion).ti,ab<br>3. Medline(marginal cord insertion).ti,ab<br>4. Medline(Velamentous cord insertion).ti,ab<br>5. Medline(bilobed placenta).ti,ab<br>6. Medline(succenturiate lobe).ti,ab<br>7. Medline(accessory lobe).ti,ab<br>8. Medline*(1 OR 2 OR 3 OR 4 OR 5 OR 6 OR 7)*
**CINAHL**
1. CINAHL(Vasa praevia OR vasa previa).ti,ab<br>2. CINAHL(abnormal cord insertion).ti,ab<br>3. CINAHL(marginal cord insertion).ti,ab<br>4. CINAHL(Velamentous cord insertion).ti,ab<br>5. CINAHL(bilobed placenta).ti,ab<br>6. CINAHL(succenturiate lobe).ti,ab<br>7. CINAHL(accessory lobe).ti,ab<br>8. CINAHL*(1 OR 2 OR 3 OR 4 OR 5 OR 6 OR 7)*
**PubMed**
1. PubMed(Vasa praevia OR vasa previa).ti,ab<br>2. PubMed(abnormal cord insertion).ti,ab<br>3. PubMed(marginal cord insertion).ti,ab<br>4. PubMed(Velamentous cord insertion).ti,ab<br>5. PubMed(bilobed placenta).ti,ab<br>6. PubMed(succenturiate lobe).ti,ab<br>7. PubMed(accessory lobe).ti,ab<br>8. PubMed*(1 OR 2 OR 3 OR 4 OR 5 OR 6 OR 7)*
**EMBASE**
1. EMBASE(Vasa praevia OR vasa previa).ti,ab<br>2. EMBASE(abnormal cord insertion).ti,ab<br>3. EMBASE(marginal cord insertion).ti,ab<br>4. EMBASE(Velamentous cord insertion).ti,ab<br>5. EMBASE(bilobed placenta).ti,ab<br>6. EMBASE(succenturiate lobe).ti,ab<br>7. EMBASE(accessory lobe).ti,ab<br>8. EMBASE*(1 OR 2 OR 3 OR 4 OR 5 OR 6 OR 7)*
