# Supplementary material for: Incidence of vasa praevia: a systematic review and meta-analysis
Source: BMJ Open. 2023 Sep 20;13(9):e075245. doi: 10.1136/bmjopen-2023-075245 (PMC10514663; doi:10.1136/bmjopen-2023-075245)
Supplement: Supplementary data [file bmjopen-2023-075245supp003.pdf]

**Supplementary table 2.** Methodological assessment of included studies based on Newcastle – Ottawa Score Summary

| Study | Selection | Comparability | Outcome | NOS Score |
|---|---|---|---|---|
| Lee *et al.*, 2000[24] | ★★★ | ★ | ★★★ | 7 |
| Catanzarite *et al.*, 2001[8] | ★★★ | ★ | ★★★ | 7 |
| Schachter *et al.*, 2002[25] | ★★★ | ★ | ★★★ | 7 |
| Baulies *et al.*, 2007[26] | ★★★ | ★ | ★★★ | 7 |
| Suzuki *et al.*, 2008[27] | ★★★ | ★ | ★★★ | 7 |
| Smorgick *et al.*, 2010[28] | ★★★ | - | ★★★ | 6 |
| Hasegawa *et al.*, 2010[29] | ★★★ | ★★ | ★★★ | 8 |
| Kanda *et al.*, 2011[30] | ★★★ | ★ | ★★★ | 7 |
| Rebarber *et al.*, 2013[31] | ★★★ | ★ | ★★★ | 7 |
| Bronsteen *et al.*, 2013[32] | ★★★ | ★ | ★★★ | 7 |
| Hasegawa *et al.*, 2015[33] | ★★★ | ★ | ★★★ | 7 |
| Catanzarite *et al.*, 2016[10] | ★★★ | ★ | ★★★ | 7 |
| Kulkarni *et al.*, 2017[34] | ★★★ | ★ | ★★★ | 7 |
| Nohuz *et al.*, 2017[35] | ★★★ | ★ | ★★★ | 7 |
| Sullivan *et al.*, 2017[11] | ★★★ | ★ | ★★★ | 7 |
| Yeaton-Massey *et al.*, 2019[36] | ★★ | ★ | ★★★ | 6 |
| Derisbourg *et al.*, 2019[37] | ★★★ | ★ | ★★★ | 7 |
| Klahr *et al.*, 2019[38] | ★★ | ★ | ★★★ | 6 |
| La *et al.*, 2020[39] | ★★★ | ★ | ★★★ | 7 |
| Zhang *et al.*, 2020[12] | ★★★ | ★ | ★★★ | 7 |
| Gross *et al.*, 2021[40] | ★★★ | ★ | ★★★ | 7 |
| Sutera *et al.*, 2021[41] | ★★★ | ★ | ★★★ | 7 |
| Liu *et al.*, 2021[42] | ★★★ | ★ | ★★★ | 7 |
| Kamijo *et al.*, 2022[43] | ★★★ | ★ | ★★★ | 7 |
