# Supplementary material for: Incidence of vasa praevia: a systematic review and meta-analysis
Source: BMJ Open. 2023 Sep 20;13(9):e075245. doi: 10.1136/bmjopen-2023-075245 (PMC10514663; doi:10.1136/bmjopen-2023-075245)
Supplement: Supplementary data [file bmjopen-2023-075245supp005.pdf]

Supplementary Table 4. Characteristics of the included studies

Author (year)	Study design	Study location	Population	Study period	VP Cases	Population screened	Characteristics and outcomes	Postnatal confirmation of VP
Baulies *et al.*, 2007[26]	Retrospective cohort study	Spain	Unselected pregnancies	2000-2005	9	12,063	Risk factors, delivery and neonatal outcomes	Yes
Rebarber *et al.*, 2013[31]	Retrospective cohort study	United States	Unselected pregnancies	2005-2012	24	27,573	Resolution rate, delivery outcomes, hospital stay	Yes
Catanzarite *et al.*, 2016[10]	Retrospective cohort study	United States	Unselected pregnancies	2003-2015	96	100,481	Risk factors, Antepartum haemorrhage, tocolysis, delivery and neonatal outcomes	Yes
Kulkarni *et al.*, 2017[34]	Retrospective cohort study	United States	Unselected pregnancies	2009-2017	35	56,000	Risk factors, Antepartum haemorrhage, delivery and neonatal outcomes	Yes
Nohuz *et al.*, 2017[35]	Retrospective cohort study	France	Unselected pregnancies	2011-2015	8	18,152	Risk factors, Antepartum haemorrhage, delivery and neonatal outcomes	Yes
Sullivan *et al.*, 2017[11]	Population cohort study	Australia	Unselected pregnancies	2013-2014	63	294,045	Risk factors, Antepartum haemorrhage, delivery and	Yes

							neonatal outcomes	
Klahr *et al.*, 2019[38]	Retrospective cohort study	United States	Unselected pregnancies	2005-2018	61	37,236	Maternal characteristics, placenta and vessel types	Yes
La *et al.*, 2020[39]	Retrospective cohort study	Australia	Unselected pregnancies	2007-2017	19	56,045	Maternal characteristics, Antepartum haemorrhage, Preterm ROM, delivery and neonatal outcomes	Yes
Zhang *et al.*, 2020[12]	Retrospective cohort study	United Kingdom	Unselected pregnancies	2011-2018	21	26,830	Maternal characteristics, delivery and neonatal outcomes	Yes
Gross *et al.*, 2021[40]	Retrospective cohort study	Austria	Unselected pregnancies	2018-2019	21	5,905	Maternal characteristics, delivery and neonatal outcomes	Yes
Sutera *et al.*, 2021[41]	Retrospective cohort study	Italy	Unselected pregnancies	2007-2018	24	89,600	Maternal characteristics, delivery and neonatal outcomes	Yes
Kamijo *et al.*, 2022[43]	Retrospective cohort study	Japan	Unselected pregnancies	2010-2020	14	8,723	Type of VP, gestation at delivery, Birth weight, neonatal Hb	Yes

VP = Vasa praevia; ROM = rupture of membranes
