# Supplementary figures and images for: Incidence of vasa praevia: a systematic review and meta-analysis
Source: BMJ Open. 2023 Sep 20;13(9):e075245. doi: 10.1136/bmjopen-2023-075245 (PMC10514663; doi:10.1136/bmjopen-2023-075245)

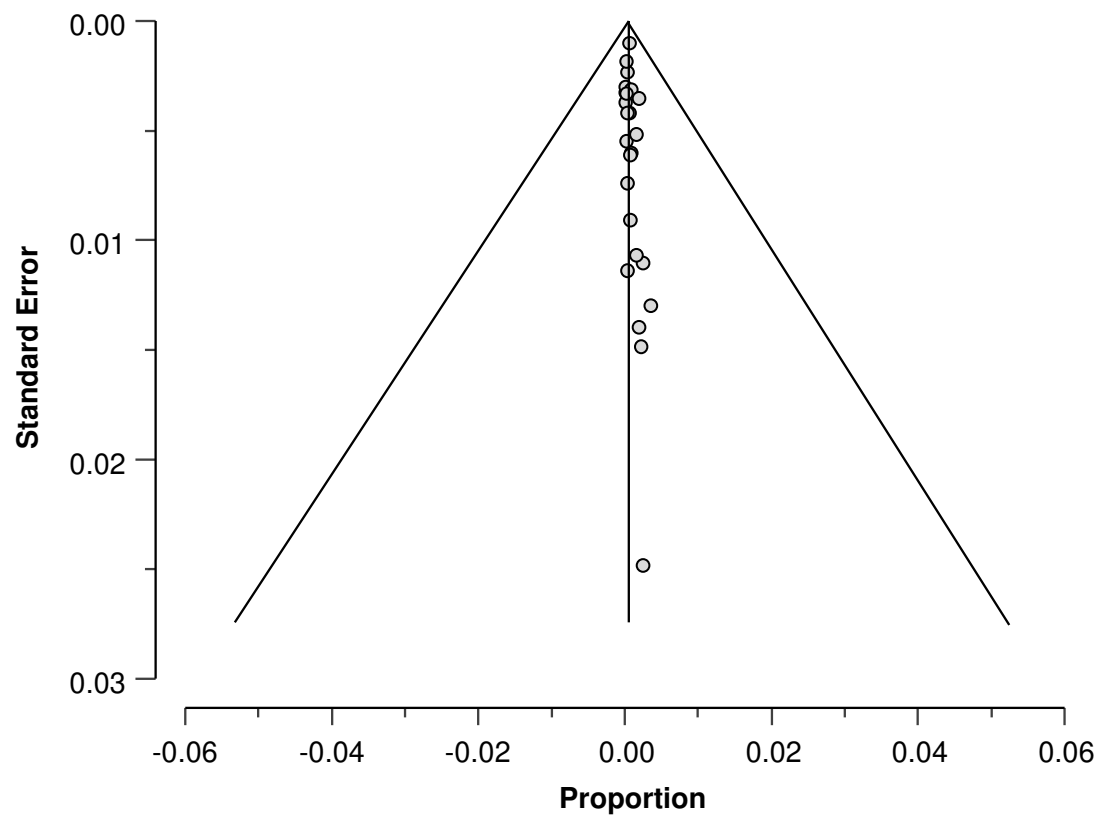

Supplementary Figure F1.

Supplement: Supplementary data [file bmjopen-2023-075245supp001.pdf]
