# Supplementary material for: Incidence of vasa praevia: a systematic review and meta-analysis
Source: BMJ Open. 2023 Sep 20;13(9):e075245. doi: 10.1136/bmjopen-2023-075245 (PMC10514663; doi:10.1136/bmjopen-2023-075245)
Supplement: Author's manuscript [file bmjopen-2023-075245.draft_revisions.pdf]

BMJ Open is committed to open peer review. As part of this commitment we make the peer review history of every article we publish publicly available.

When an article is published we post the peer reviewers' comments and the authors' responses online. We also post the versions of the paper that were used during peer review. These are the versions that the peer review comments apply to.

The versions of the paper that follow are the versions that were submitted during the peer review process. They are not the versions of record or the final published versions. They should not be cited or distributed as the published version of this manuscript.

BMJ Open is an open access journal and the full, final, typeset and author-corrected version of record of the manuscript is available on our site with no access controls, subscription charges or pay-per-view fees (http://bmjopen.bmj.com).

If you have any questions on BMJ Open's open peer review process please email info.bmjopen@bmj.com

Incidence of Vasa praevia: - A systematic review and meta-analysis

Journal:	*BMJ Open*
Manuscript ID	bmjopen-2023-075245
Article Type:	Original research
Date Submitted by the Author:	01-May-2023
Complete List of Authors:	Zhang, Weiyu; Medway NHS Foundation Trust, Medway Fetal and Maternal Medicine Centre<br>Giacchino, Tara; Medway NHS Foundation Trust, Medway Fetal and Maternal Medicine Centre<br>Chanyarungrojn, Pannapat Amy; Medway NHS Foundation Trust, Medway Fetal and Maternal Medicine Centre<br>Ionescu, Olivia; Medway NHS Foundation Trust, Medway Fetal and Maternal Medicine Centre<br>Akolekar, Ranjit; Canterbury Christ Church University, Institute of Medical Sciences; Medway NHS Foundation Trust, Medway Fetal and Maternal Medicine Centre
Keywords:	Prenatal diagnosis < OBSTETRICS, Fetal medicine < OBSTETRICS, Systematic Review

SCHOLARONE™
Manuscripts

I, the Submitting Author has the right to grant and does grant on behalf of all authors of the Work (as defined in the below author licence), an exclusive licence and/or a non-exclusive licence for contributions from authors who are: i) UK Crown employees; ii) where BMJ has agreed a CC-BY licence shall apply, and/or iii) in accordance with the terms applicable for US Federal Government officers or employees acting as part of their official duties; on a worldwide, perpetual, irrevocable, royalty-free basis to BMJ Publishing Group Ltd ("BMJ") its licensees and where the relevant Journal is co-owned by BMJ to the co-owners of the Journal, to publish the Work in this journal and any other BMJ products and to exploit all rights, as set out in our licence.

The Submitting Author accepts and understands that any supply made under these terms is made by BMJ to the Submitting Author unless you are acting as an employee on behalf of your employer or a postgraduate student of an affiliated institution which is paying any applicable article publishing charge ("APC") for Open Access articles. Where the Submitting Author wishes to make the Work available on an Open Access basis (and intends to pay the relevant APC), the terms of reuse of such Open Access shall be governed by a Creative Commons licence – details of these licences and which Creative Commons licence will apply to this Work are set out in our licence referred to above.

Other than as permitted in any relevant BMJ Author's Self Archiving Policies, I confirm this Work has not been accepted for publication elsewhere, is not being considered for publication elsewhere and does not duplicate material already published. I confirm all authors consent to publication of this Work and authorise the granting of this licence.

For peer review only

Title: Incidence of Vasa praevia: - A systematic review and meta-analysis

Authors: Weiyu Zhang[1], Tara Giacchino[1], Pannapat Amy Chanyarungrojn[1], Olivia Ionescu[1], Ranjit Akolekar[1,2]

Key words: Vasa praevia, Incidence, Stillbirths, Prenatal diagnosis

Short Title: Incidence of vasa praevia

Affiliation:

1. Medway Fetal and Maternal Medicine Centre, Medway Maritime Hospital, United Kingdom
2. Institute of Medical Sciences, Canterbury Christ Church University, Rowan William's Court, Kent, United Kingdom

Corresponding author:

Professor R Akolekar, MD, MRCOG
Institute of Medical Sciences,
Canterbury Christ Church University,
Rowan William's Court,
Chatham, Kent ME4 4UF
Email: ranjit.akolekar@canterbury.ac.uk

Word count: 2,730

For peer review only - http://bmjopen.bmj.com/site/about/guidelines.xhtml

ABSTRACT

Objectives: To derive accurate estimates of the incidence of vasa praevia (VP) in a routine population of unselected pregnancies

Design: Systematic review and meta-analysis

Data sources: A search of MEDLINE, EMBASE, CINHAL and the Cochrane database was performed to review relevant citations reporting outcomes in pregnancies with VP from January 2000 till 5th April 2023.

Eligibility criteria for selection of studies: Prospective or retrospective cohort or population studies that provided data regarding VP cases in routine unselected pregnancies during the study period. We included studies published in the English language after the year 2000 to reflect contemporary obstetric and neonatal practice.

Data extraction and synthesis: Two reviewers independently screened the retrieved citations and extracted data. The methodological quality of studies was assessed using the Newcastle-Ottawa Scale (NOS) and quality of systematic review was validated with PRISMA (Preferred Reporting Items for Systematic reviews and Meta-Analyses).

Results: A total of 3,847 citations were screened and 82 full-text manuscripts were retrieved for analysis. There were 24 studies that met inclusion criteria, of which 12 studies reported prenatal diagnosis with a systematic protocol of screening. There were 1,320 pregnancies with VP in a total population of 2,278,561 pregnancies; the weighted pooled incidence of VP was 0.79 (95%CI: 0.59-1.01) per 1,000 pregnancies, corresponding to 1 case of VP per 1,271 (95% CI: 990-1,692) pregnancies. Nested sub-analysis of studies reporting screening for VP based on a specific protocol identified 395 pregnancies with VP in a population of 732,654 pregnancies with weighted pooled incidence of 0.82 (95%CI: 0.53-1.18) per 1,000 pregnancies (1 case of VP per 1,218 (95% CI: 847-1,901) pregnancies.

Conclusion: The incidence of VP in unselected pregnancies is 1 in 1,218 pregnancies. Incorporation of strategies to screen for VP in routine clinical practice is likely to prevent 5-6% of stillbirths.

PROSPERO registration number: CRD42020125495.

For peer review only

STRENGTHS AND LIMITATIONS OF THE STUDY

- This systematic review and meta-analysis provides an accurate estimate of the true incidence of vasa praevia in a routine population of unselected pregnancies.
- Unlike previous studies, we only included observational cohort studies that provided data regarding vasa praevia and corresponding group of control pregnancies delivered during the study period rather than including case-control studies and those studies that were carried out on high-risk populations only.
- We included studies that were published since the year 2000 to reflect contemporary clinical practice.
- We undertook a sub-group nested analysis in studies that reported a systematic and specific policy of screening for vasa praevia in their hospital and reported both cases of vasa praevia as well as the population of control pregnancies.
- We mitigated the heterogeneity between studies by standardising the inclusion of studies for analysis, use of a random effects model to allow for variance between studies and undertaking a regression analysis to investigate factors affecting incidence.

For peer review only

For peer review only - http://bmjopen.bmj.com/site/about/guidelines.xhtml

INTRODUCTION

Vasa praevia (VP) is defined as presence of fetal blood vessels, arterial or venous, unsupported by placental tissue or umbilical cord traversing the amniotic membranes in the lower uterine segment in close proximity to the internal cervical os.[1-5] These unsupported fetal vessels can be damaged either during the antenatal period or in labour leading to severe hypovolemic shock and haemorrhagic fetal death.[4,6,7] There is considerable evidence suggesting that prenatal diagnosis of VP improves perinatal survival and lack of antenatal detection is associated with a high-risk of stillbirths, neonatal deaths and an increased risk of hypoxic morbidity in the survivors.[9-13] Despite the compelling evidence in favour of prenatal diagnosis in improving pregnancy outcomes in pregnancies with VP, there are currently no recommendations for screening for VP in routine clinical practice.[14] The United Kingdom (UK) National Screening Committee examined the evidence with regard to screening for VP and reported that there was low volume of evidence on the epidemiology of VP or its outcomes in the UK to justify screening.[15]

A systematic review examined the incidence of VP and reported that there was a huge variation in studies reporting VP incidence ranging from 0.19-2.21 per 1,000 pregnancies (1 per 452 to 1 per 5,882 pregnancies) with a mean estimated incidence of 0.6 per 1,000 pregnancies (1 per 1,666 pregnancies).[16] This systematic review had significant heterogeneity and variation as it included not just cohort studies but case-control studies and those from high-risk populations. The estimated incidence in this systematic review is likely to have been underestimated as many of the included studies reported data from 80s and 90s when VP was more likely to be an incidental rather than an intentional diagnosis. In a recent large prospective observational screening study of more than 26,000 pregnancies, we demonstrated the impact of routine screening for VP based on a 2-stage screening strategy and reported that the incidence of VP was about 1 in 1,277 pregnancies.[12]

The objective of this study was to undertake a systematic review of literature and undertake a meta-analysis to estimate an accurate incidence of VP in a routine population of unselected pregnancies based on analysis of studies that reported a policy of routinely screening for VP.

METHODS

<u>Data sources and search strategy</u>

This systematic review and meta-analysis was undertaken based on an *a-priori* designed study protocol recommended for systematic reviews and meta-analyses.[17] The study protocol for the

For peer review only - http://bmjopen.bmj.com/site/about/guidelines.xhtml

systematic review was registered in advance with the PROSPERO International prospective register of systematic reviews (Registration number: CRD42020125495) as a study to investigate perinatal outcomes as well as incidence of VP. An electronic search of MEDLINE, EMBASE and The Cochrane Library was carried out on 30th October 2022 and repeated on 5th April 2023 utilising combinations of the relevant Medical Subject Heading (MeSH) terms, key words and word variants for “vasa praevia”, “abnormal cord insertion”, “velamentous cord”, marginal cord”, “bilobed placenta” and “succenturiate lobe”. The search and selection criteria were restricted to studies reported in the English language.

Selection criteria for studies and data extraction

The studies eligible for inclusion in this study were prospective or retrospective cohort studies and population-based studies reporting cases with VP in which the authors reported data about the total screened population during the study period. We only included studies published after 2000 to reflect contemporary clinical practice. We did a nested sub-analysis for studies which reported in their methodology that they had a routine screening policy for assessing umbilical cord insertion and VP. The citations were examined by two reviewers (WZ and PC) to produce a list of relevant studies after exclusion of duplicates, those that did not fit selection criteria after review of title and abstract, those that were case-reports, letter to editors, review articles or conference abstracts and those that were case series or case-control studies. The reference lists of relevant articles were searched, and any inconsistencies were resolved by discussion with a third reviewer (RA).

The citations retrieved following this search strategy were examined for relevance to this study to investigate incidence based on above eligibility criteria and in each study, we extracted information about authors, study location, years of enrolment for cases and controls, study design, sample size, whether the study was single or multi-centre, whether the study was in high-risk population or unselected general pregnant population, and whether there was a routine policy of screening for umbilical cord insertion and VP during the study period in the reported studies. Data extracted for each study was inputted into contingency tables.

Quality Assessment

The methodological quality of studies included in the review was assessed using the Newcastle-Ottawa Scale (NOS).[18] The quality of this systematic review and meta-analysis was validated with PRISMA (Preferred Reporting Items for Systematic reviews and Meta-Analyses). The PRISMA statement for this study included a checklist and a flow chart to allow uniform and transparent reporting of the systematic review and meta-analysis.[19]

For peer review only - http://bmjopen.bmj.com/site/about/guidelines.xhtml

Estimation of summary statistics for incidence of vasa praevia (VP)

Data were entered in contingency tables for cases and control pregnancies to calculate weighted pooled incidence (95% confidence intervals [CI]). The incidence of VP was reported as total number of cases per 1,000 pregnancies as well as total number of pregnancies for every 1 case of VP. Summary statistics (95%CI) from each study were then combined to obtain a pooled estimate which was calculated as a weighted average of the individual study estimates. The pooled summary statistics were estimated using random-effects model (REM) to allow for assessment of between-study variability in results by weighting studies using a combination of their own variance as well as the between-study variance.[20] The heterogeneity between studies was estimated using Cochrane's heterogeneity statistic Q, and the I^2 statistic. Regression analysis was undertaken to examine factors providing a significant contribution to the prediction of incidence of VP. We also estimated the potential impact of introducing a policy for screening for VP in preventing stillbirths in the UK based on annual number of total births and stillbirths reported by the Office of National Statistics, National Records of Scotland and Northern Ireland Statistics and Research Agency.[21-23] The statistical software package StatsDirect version 2.7.9 (StatsDirect Ltd, Cheshire, United Kingdom) and MedCalc Statistical Software version 16.4.3 (MedCalc Software, Ostend, Belgium) were used for data analysis.

Patient and public involvement

No patients were involved in setting the research question, in developing plans for design, interpretation, reporting or implementation of the study.

RESULTS

Data search results

The electronic search from the databases yielded 3,847 potential citations; of these, we excluded 1,671 citations as they were duplicates, 1,504 after review of study title and 590 citations after review of the abstract. We retrieved 82 manuscripts in full text for detailed assessment and excluded a further 58 studies that did not meet the selection criteria, thus finally including 24 studies for systematic review and meta-analysis. The search criteria are outlined in supplementary table S1, and the study selection process is outlined in the flow chart in Figure 1.

Characteristics of studies included in the systematic review

There were 24 studies[8,10-12,24-43] included in the systematic review, which provided data regarding incidence of VP. There were 7 cohort studies published in the first decade (2000 to

For peer review only - http://bmjopen.bmj.com/site/about/guidelines.xhtml

2010)[8,24-29] and 17 studies in the second decade (2011 to 2020)[10-12,30-43]. The published studies were from across different geographical areas; 8 studies from United states[8,10,24,31,32,34,36,38] 5 studies from Japan[27,29,30,33,43], 6 from Europe[12,26,35,37,40,41], 2 each from Australia[11,39] and Israel[25,28] and 1 from China[42]. All 24 studies reported the total screened population during the study period and the number of cases of VP detected, thus providing a basis of estimating incidence of VP. Screening studies which report a policy-based assessment of an unselected cohort of pregnancies are more likely to reflect the true incidence of VP, compared to studies that included an incidental diagnosis of VP. Therefore, we identified studies that described a specific protocol for assessment of all pregnancies during the study period for prenatal diagnosis of VP after excluding those that had a variable protocol for identification of at-risk pregnancies, those that were carried out in a specific high-risk population such as those with velamentous cord insertion,[37] succenturiate lobe,[27] *in vitro* fertilisation pregnancies[25] or those who were only delivered by caesarean section.[36] After exclusion of above studies, we identified 12 studies,[10-12,26,31,34,35,38-41,43] in which the authors reported that they had a systematic protocol to document the placental location and umbilical cord insertion at the first or second trimester scan; those that were suspected to have VP were further scanned with transvaginal ultrasound to confirm the diagnosis.

<u>Assessment of quality and heterogeneity of studies</u>

The methodological quality of studies included in this systematic review was assessed using the NOS. The rating of the included studies according to the NOS based on selection, comparability and outcome are shown in Supplementary table S2. The PRISMA guidance was followed for reporting this meta-analysis and is reported in Supplementary table S3). The funnel plot of included studies demonstrating the publication bias is shown in Supplementary figure F1.

<u>Incidence of vasa praevia (VP)</u>

There were 24 studies that reported data about cases with VP with a corresponding cohort of total number of unselected pregnancies screened in a routine population during the study period.[8,10-12,24-43] There were 1,320 pregnancies with VP in a total population of 2,278,561 pregnancies. The weighted pooled incidence of VP was 0.79 (95%CI: 0.59-1.01) per 1,000 pregnancies screened, which corresponded to an incidence of 1 case of VP per 1,271 (95% CI: 990-1,692) pregnancies (Table 1).

There were 12 studies that reported data regarding incidence of VP along with the total pregnancies screened based on a specific screening protocol.[10-12,26,31,34,35,38-41,43] There were 395 pregnancies with VP in a total population of 732,654 pregnancies. The weighted pooled

For peer review only - http://bmjopen.bmj.com/site/about/guidelines.xhtml

incidence of VP was 0.82 (95%CI: 0.53-1.18) per 1,000 pregnancies screened, which corresponded to an incidence of 1 case of VP per 1,218 (95% CI: 847-1,901) pregnancies (Table 2, Figure 2).

Regression analysis demonstrated that in prediction of the incidence of VP, there was a significant independent contribution from the publication date of the study, whether the study was published in the first decade (2000-2010) or in the second decade (2011-2022) ($p<0.011$) but there was no significant association with the country in which the study was conducted ($p=0.408$) ($R^2=0.208$; $p<0.05$). There was a significant increase in incidence of VP from studies published in the second decade (2011-2022) compared to first decade (2000-2010) (OR: 2.60; 95%CI: 2.08-3.25) ($p<0.01$).

Potential impact on prevention of stillbirths in United Kingdom

There were total of 697,551 births in the UK with 2,866 (0.41%) stillbirths in 2021. Given an incidence of VP of 1 in 1,218 pregnancies, there would be an estimated 573 pregnancies with VP per year in the UK. Based on estimates from the systematic review and meta-analysis reporting perinatal survival in pregnancies with VP, the livebirth rate in pregnancies with VP with and without prenatal diagnosis is 98.6% (95%CI: 96.7-99.7) and 72.1% (95%CI: 50.6-89.4), respectively; therefore, the expected number of stillbirths in pregnancies with VP in those with and without prenatal diagnosis would be 8 (95%CI: 2-19) and 160 (95% CI: 61-283). Therefore, the potential impact of introducing a policy of screening for VP in clinical practice could contribute to prevention of 5.58% (95%CI: 2.13-9.87) of stillbirths in the UK.

DISCUSSION

Principal findings of the study

The findings of our systematic review and meta-analysis demonstrate that the incidence of VP in an unselected population of pregnancies is 1 in 1,218 pregnancies. The estimated incidence of VP in our study is based on weighted pooled estimates calculated firstly, from meta-analysis of studies published in the last 20 years to reflect contemporary obstetric and neonatal practice and secondly, by inclusion of those studies that reported a specific policy of assessment of placental location and umbilical cord insertion for prenatal diagnosis of VP in their routine practice. The incidence of VP was not affected by the study location or where the study was carried out, reflecting that in centres that have a specific protocol for assessment of VP as described above, there is no significant variation in reported incidence. Our study

For peer review only - http://bmjopen.bmj.com/site/about/guidelines.xhtml

demonstrates that if screening for prenatal diagnosis of VP was introduced in routine clinical practice, it can potentially prevent 5-6% of stillbirths.

Comparison with previous studies

The results of our study demonstrate that VP is not an uncommon obstetric complication and has an incidence of 1 in 1,218 pregnancies, which is higher than that reported in previous studies.[16] A previous systematic review reported a wide variation in the incidence of VP from 1 per 452 to 5,882 pregnancies with a mean incidence of 1 in 1,666 pregnancies. The authors included a wide range of studies including case-control studies, retrospective cohort studies, studies carried out on high-risk populations (such as those with pregnancies conceived with *in vitro* fertilisation, studies examining outcomes in pregnancies with bilobed placenta or in those that had a velamentous cord insertion), those that reported an incidental diagnosis of VP rather than cases detected in an unselected population based on a systematic protocol of screening and inclusion of studies that did not report whether there was postnatal confirmation of prenatal findings of VP, thus leading to a wide heterogeneity in the results and potentially underestimation of the incidence. Moreover, the authors included studies reporting data from 80s and 90s, which are more likely to be incidentally diagnosed cases of VP rather than as a result of intentional screening.

Implications for clinical practice

There is robust and unequivocal evidence from several studies, including our own prospective cohort screening study, suggesting that prenatal diagnosis of VP is associated with high rates of livebirths and perinatal survival.[10-12,30,34,35,40-42] A recent systematic review and meta-analysis summarised and confirmed the results from previous studies demonstrating that prenatal diagnosis of VP is associated with a 99% rate of perinatal survival but a lack of an antenatal diagnosis increases the risk of a perinatal hypoxic morbidity 50-fold and that of perinatal death by 25-fold.[13] There are few other examples in obstetric practice which demonstrate such a significant and stark impact of prenatal diagnosis on improving pregnancy outcomes such as the prenatal diagnosis of VP. There are 12 relatively recent studies[10-12,26,31,34,35,38-41,43] which report that systematic and routine screening for VP is feasible in routine clinical practice; in fact, our recent prospective observational study of more than 26,000 pregnancies demonstrates that a 2-stage screening strategy for VP can be easily implemented in routine clinical practice and such a policy is associated with a high rate of prenatal diagnosis and good perinatal outcomes.[12]

Strengths and limitations

The strength of our study is that it summarises results of all studies reported in the last two decades and provides accurate summary estimates for incidence of VP in current obstetric

For peer review only - http://bmjopen.bmj.com/site/about/guidelines.xhtml

practice. We used a standardised methodology for conducting this systematic review and meta-analysis with design of an *a-priori* protocol, registration with the international register at PROSPERO, a comprehensive search strategy, appropriate quality assessment of studies included in systematic review with NOS and validation of systematic review with PRISMA checklist. The limitations of our study relate to standard biases associated with meta-analysis such as inclusion of studies with different sample size, methodology and design which may introduce heterogeneity in the analysis. However, as a means to minimize the effects of such variation and heterogeneity, we reviewed all these studies in detail to select and identify those studies that provide data from low-risk unselected pregnancies rather than high-risk populations. This allowed us to do a nested meta-analysis to estimate an accurate weighted pooled summary statistic, which is more reflective of true population incidence rather than just a pooled estimated from all reported studies. We used a random effects model to mitigate the impact of heterogeneity as this model allows for assessment of between-study variability in results by weighing studies using a combination of their own variance as well as the between-study variance. Furthermore, we performed regression analyses to examine temporal and spatial trends in VP incidence such as the publication dates as well as the location of where the studies were carried out.

Conclusions

VP is not an uncommon obstetric complication. The incidence of VP in an unselected population is 1 in 1,218 pregnancies, which is considerably higher than previously reported estimates. Further research studies should be undertaken to model and investigate strategies for incorporating prenatal screening for VP in routine clinical practice, given the feasibility of screening and major impact on prevention of stillbirths and hypoxic morbidity with prenatal diagnosis of VP.

Acknowledgement: We are grateful to the Medway Library and Knowledge Service for their help and assistance with search strategy. This study is a part of the PhD thesis of Weiyu Zhang for the Canterbury Christ Church University.

Author statement: WZ, PC, TG and OI contributed to literature search, study selection, data extraction and assessment of study quality. WZ and RA wrote the initial and final draft of the manuscript. RA conceived the study and supervised the data extraction, discussed studies for inclusion following initial review and agreed the final draft of the manuscript for submission. All authors reviewed the initial and final draft of the manuscript and contributed to the intellectual content.

For peer review only - http://bmjopen.bmj.com/site/about/guidelines.xhtml

Data availability statement: Data are available upon reasonable request.

Ethics approval: No ethical approval was required for this study.

Conflict of interest: None

Funding: No funding was received for this study.

For peer review only

For peer review only - http://bmjopen.bmj.com/site/about/guidelines.xhtml

REFERENCES

1. McNair AJ. Placenta praevia, with vasa praevia; Caesarean section. *Proc R Soc Med* 1921; 14: 195-196.
2. Alment EA. Vasa praevia simulating antepartum hemorrhage. *Br Med J* 1949; 2: 1273.
3. Sinkey RG, Odibo AO, Dashe JS. Diagnosis and management of vasa previa. Society of Maternal-Fetal (SMFM) Publications Committee. *Am J Obstet Gynecol* 2015; 213: 615-619.
4. Oyelese KO, Turner M, Lees C, Campbell S. Vasa previa: an avoidable obstetric tragedy. *Obstet Gynecol Surv* 1999; 54: 138–45.
5. Catanzarite V, Oyelese Y. Diagnosis and management of vasa previa. *Am J Obstet Gynecol* 2016; 214: 764.
6. Robert JA, Sepulveda W. Fetal exsanguination from ruptured vasa previa: still a catastrophic event in modern obstetrics. *J Obstet Gynaecol* 2003; 23: 574.
7. Antoine C, Young BK, Silverman F, Greco MA,lvarez SP. Sinusoidal fetal heart rate pattern with vasa previa in twin pregnancy. *J Reprod Med* 1982; 27: 295–300.
8. Catanzarite V, Maida C, Thomas W, Mendoza A, Stanco L, Piacquadio KM. Prenatal sonographic diagnosis of vasa previa: ultrasound findings and obstetric outcome in ten cases. *Ultrasound Obstet Gynecol* 2001; 18:109-115.
9. Oyelese Y, Catanzarite V, Prefumo F, Lashley S, Schachter M, Tovbin Y, Goldstein V, Smulian JC. Vasa previa: the impact of prenatal diagnosis on outcomes. *Obstet Gynecol* 2004; 103: 937-942.
10. Catanzarite V, Cousins L, Daneshmand S, Schwendemann W, Casele H, Adamczak J, Tith T, Patel A. Prenatally Diagnosed Vasa Previa: A Single-Institution Series of 96 Cases. *Obstet Gynecol* 2016; 128: 1153-1161.
11. Sullivan EA, Javid N, Duncombe G, Li Z, Safi N, Cincotta R, Homer CSE, Halliday L, Oyelese Y. Vasa Previa Diagnosis, Clinical Practice, and Outcomes in Australia. *Obstet Gynecol* 2017; 130: 591-598.
12. Zhang W, Geris S, Beta J, Ramadan G, Nicolaides KH, Akolekar R. Prevention of stillbirth: impact of two-stage screening for vasa previa. *Ultrasound Obstet Gynecol*. 2020; 55: 605-612.
13. Zhang W, Geris S, Al-Emara N, Ramadan G, Sotiriadis A, Akolekar R. Perinatal survival in pregnancies with a prenatal diagnosis of vasa praevia: a systematic review and meta-analysis. *Ultrasound Obstet Gynecol*. 2021; 57: 710-719.
14. Jauniaux E, Alfirevic Z, Bhide AG, Burton GJ, Collins SL, Silver R; Royal College of Obstetricians and Gynaecologists. Vasa Praevia: Diagnosis and Management: Green-top Guideline No. 27b. *BJOG*. 2019; 126: e49-e61.

For peer review only - http://bmjopen.bmj.com/site/about/guidelines.xhtml

15. Screening for Vasa praevia: An evidence map to outline the volume and type of evidence related to screening for vasa praevia for the UK National Screening Committee: Version 1.1: Costello Medical: UK National Screening Committee. March 2023. Vasa praevia - UK National Screening Committee (UK NSC) - GOV.UK (view-health-screening-recommendations.service.gov.uk). Accessed 28th April 2023.

16. Ruiter L, Kok N, Limpens J, Derks JB, de Graaf IM, Mol B, Pajkrt E. Incidence of and risk indicators for vasa praevia: a systematic review. *BJOG.* 2016; 23: 1278-87.

17. NHS Centre for Reviews and Dissemination. Systematic reviews: CRD's guidance for undertaking reviews in health care. University of York: York, UK, 2009.

18. Wells GA, Shea B, O'Connell D, Peterson J, Welch V, Losos M, Tugwell P. The Newcastle-Ottawa Scale (NOS) for assessing the quality of nonrandomized studies in meta-analysis. http://www.ohri.ca/programs/clinical_epidemiology/oxford.asp.

19. Moher D, Liberati A, Tetzlaff J, Altman DG, The PRISMA Group. Preferred Reporting Items for Systematic Reviews and Meta-Analyses: The PRISMA Statement. *BMJ* 2009; 39: b2535.

20. DerSimonian R, Laird N: Meta-analysis in clinical trials. *Control Clin Trials* 1986; 7: 177-188.

21. Births in England and Wales: Summary tables: August 2022. https://www.ons.gov.uk/peoplepopulationandcommunity/birthsdeathsandmarriages/livebirths/bulletins/birthsummarytablesenglandandwales/2021. Accessed 28th April 2023.

22. Vital events reference tables. National records of Scotland. June 2022. https://www.nrscotland.gov.uk/statistics-and-data/statistics/statistics-by-theme/vital-events/general-publications/vital-events-reference-tables/2021. Accessed 28th April 2023.

23. Stillbirths: Northern Ireland Statistics and Research Agency. November 2022. https://www.nisra.gov.uk/publications/stillbirth-and-infant-death-numbers. Accessed 28th April 2023.

24. Lee W, Lee VL, Kirk JS, Sloan CT, Smith RS, Comstock CH. Vasa previa: prenatal diagnosis, natural evolution, and clinical outcome. *Obstet Gynecol* 2000; 95: 572-576.

25. Schachter M, Tovbin Y, Arieli S, Friedler S, Ron-El R, Sherman D. In vitro fertilisation is a risk factor for vasa praevia. *Fertil Steril*. 2002; 78:642-643.

26. Baulies S, Maiz N, Muñoz A, Torrents M, Echevarría M, Serra B. Prenatal ultrasound diagnosis of vasa praevia and analysis of risk factors. *Prenat Diagn* 2007; 27: 595-599.

27. Suzuki S, Igarashi M. Clinical significance of pregnancies with succenturiate lobes of placenta. *Arch Gynecol Obstet*. 2008; 277:299-301.

For peer review only

For peer review only - http://bmjopen.bmj.com/site/about/guidelines.xhtml

28. Smorgick N, Tovbin Y, Ushakov F, Vaknin Z, Barzilay B, Herman A, Maymon R Is neonatal risk from vasa previa preventable? The 20-year experience from a single medical center. *J Clin Ultrasound* 2010; 38: 118-22.

29. Hasegawa J, Farina A, Nakamura M, Matsuoka R, Ichizuka K, Sekizawa A, Okai T. Analysis of the ultrasonographic findings predictive of vasa previa. *Prenat Diagn*. 2010; 30: 1121-1125.

30. Kanda E, Matsuda Y, Kamitomo M, Maeda T, Mihara K, Hatae M. Prenatal diagnosis and management of vasa previa: a 6-year review. *J Obstet Gynaecol Res* 2011; 37: 1391-1396.

31. Rebarber A, Dolin C, Fox NS, Klauser CK, Saltzman DH, Roman AS. Natural history of vasa previa across gestation using a screening protocol. *J Ultrasound Med* 2014; 33: 141-147.

32. Bronsteen R, Whitten A, Balasubramanian M, Lee W, Lorenz R, Redman M, Goncalves L, Seubert D, Bauer S, Comstock C. Vasa previa: clinical presentations, outcomes, and implications for management. *Obstet Gynecol*. 2013; 122: 352-357.

33. Hasegawa J, Arakaki T, Ichizuka K, Sekizawa A. Management of vasa previa during pregnancy. *J Perinat Med*. 2015; 43: 783-784.

34. Kulkarni A, Powel J, Aziz M, Shah L, Lashley S, Benito C, Oyelese Y. Vasa Previa: prenatal diagnosis and outcomes: thirty-five cases from a single maternal-fetal medicine practice. *J Ultrasound Med* 2018; 37: 1017-1024.

35. Nohuz E, Boulay E, Gallot D, Lemery D, Vendittelli F. Can we perform a prenatal diagnosis of vasa previa to improve its obstetrical and neonatal outcomes? *J Gynecol Obstet Hum Reprod*. 2017; 46: 373-377.

36. Yeaton-Massey A, Girsen AI, Mayo JA, Blumenfeld YJ, El-Sayed YY, Stevenson DK, Shaw GM. Vasa previa and extreme prematurity: a population-based study. *J Perinatol. 2019;* 39:475-480.

37. Derisbourg S, Boulay A, Lamy C, Barlow P, Van Rysselberge M, Thomas D, Rozenberg S, Daelemans C. First trimester ultrasound prediction of velamentous cord insertions: a prospective study. *J Matern Fetal Neonatal Med. 2021*; 34:2642-2648.

38. Klahr R, Fox NS, Zafman K, Hill MB, Connolly CT, Rebarber A. Frequency of spontaneous resolution of vasa previa with advancing gestational age. *Am J Obstet Gynecol. 2019*; 221:646.e1-646.e7.

39. La S, Melov SJ, Nayyar R. Are we over-diagnosing vasa praevia? The experience and lessons learned in a tertiary centre. *Aust N Z J Obstet Gynaecol. 2021*; 61:217-222.

40. Gross A, Markota Ajd B, Specht C, Scheier M. Systematic screening for vasa previa at the 20-week anomaly scan. *Acta Obstet Gynecol Scand. 2021;* 100:1694-1699.

For peer review only

For peer review only - http://bmjopen.bmj.com/site/about/guidelines.xhtml

41. Sutera M, Garofalo A, Pilloni E, Parisi S, Alemanno MG, Menato G, Sciarrone A, Viora E. Vasa previa: when antenatal diagnosis can change fetal prognosis. *J Perinat Med. 2021*; 49: 915-922.

42. Liu N, Hu Q, Liao H, Wang X, Yu H. Vasa previa: Perinatal outcomes in singleton and multiple pregnancies*. Biosci Trends. 2021*; 11:15(2):118-125.

43. Kamijo K, Miyamoto T, Ando H, Tanaka Y, Kikuchi N, Shinagawa M, Yamada S, Asaka R, Fuseya C, Ohira S, Shiozawa T. Clinical characteristics of a novel "Type 3" vasa previa: case series at a single center. *J Matern Fetal Neonatal Med. 2022*; 35:7730-7736.

For peer review only

Figure Legends

Figure 1. Flow chart demonstrating process of selection of studies included in the systematic review

Figure 2. Forest plot demonstrating incidence of vasa praevia in studies reporting results of routine screening in low-risk unselected pregnancies. Incidence (%; 95% confidence intervals) is reported as number of cases per 1,000 pregnancies.

For peer review only

For peer review only - http://bmjopen.bmj.com/site/about/guidelines.xhtml

Table 1. Results from meta-analysis using a random effects model to estimate summary statistics for incidence (95% confidence interval) of vasa praevia (VP) based on studies that reported VP cases along with data regarding an unselected cohort of pregnancies during the study period

Author (year)	n/N	n/1,000 pregnancies	1 per *n* pregnancies
Lee *et al.*, 2000[24]	18/93,874	0.19 (0.11-0.30)	5,208 (3,300-8,772)
Catanzarite *et al.*, 2001[8]	11/33,208	0.33 (0.17-0.59)	3,201 (1,686-6,061)
Schachter *et al.*, 2002[25]	12/72,818	0.17 (0.09-0.29)	6,061 (3,472-11,737)
Baulies *et al.*, 2007[26]	9/12,063	0.75 (0.34-1.42)	1,340 (706-2,933)
Suzuki *et al.*, 2008[27]	3/7,713	0.39 (0.08-1.14)	2,571 (877-12,469)
Smorgick *et al.*, 2010[28]	19/110,684	0.17 (0.10-0.27)	5,814 (3,731-9,709)
Hasegawa *et al.*, 2010[29]	10/4,532	2.21 (1.06-4.05)	452 (247-943)
Kanda *et al.*, 2011[30]	10/5,131	1.95 (0.94-3.58)	513 (279-1,070)
Rebarber *et al.*, 2013[31]	24/27,573	0.87 (0.56-1.29)	1,149 (775-1,792)
Bronsteen *et al.*, 2013[32]	74/182,554	0.41 (0.32-0.51)	2,469 (1,965-3,145)
Hagesawa *et al.*, 2015[33]	21/8,176	2.57 (1.59-3.92)	389 (255-629)
Catanzarite *et al.*, 2016[10]	96/100,481	0.96 (0.77-1.17)	1,047 (857-1,292)
Kulkarni *et al.*, 2017[34]	35/56,000	0.63 (0.44-0.87)	1,600 (1,151-2,299)
Nohuz *et al.*, 2017[35]	8/18,152	0.44 (0.19-0.87)	2,268 (1,152-5,263)
Sullivan *et al.*, 2017[11]	63/294,045	0.21 (0.17-0.27)	4,673 (3,650-6,061)
Yeaton-Massey *et al.*, 2019[36]	586/945,950	0.62 (0.57-0.67)	1,616 (1,488-1,754)
Derisbourg *et al.*, 2019[37]	4/1,620	2.47 (0.67-6.31)	405 (158-1,486)
Klahr *et al.*, 2019[38]	61/37,236	1.64 (1.25-2.10)	610 (476-800)
La *et al.*, 2020[39]	19/56,045	0.34 (0.20-0.53)	2,950 (1,890-4,902)
Zhang *et al.*, 2020[12]	21/26,830	0.78 (0.49-1.20)	1,277 (836-2,062)
Gross *et al.*, 2021[40]	21/5,905	3.56 (2.20-5.43)	281 (184-455)
Sutera *et al.*, 2021[41]	24/89,600	0.27 (0.17-0.40)	3,731 (2,506-5,814)
Liu *et al.*, 2021[42]	157/79,647	1.97 (1.68-2.30)	508 (435-595)
Kamijo *et al.*, 2022[43]	14/8,723	1.60 (0.88-2.69)	625 (372-1,139)
**Pooled analysis**	**1,320/2,278,561**	**0.79 (0.59-1.01)**	**1,271 (990-1,692)**
Cochrane Q (DF)	550.11 (23)		
I^2 (inconsistency) (%) (95%CI)	95.8 (94.7-96.7)		
Egger bias (p value)	2.1799 (0.1692)		

For peer review only

For peer review only - http://bmjopen.bmj.com/site/about/guidelines.xhtml

Table 2. Results from meta-analysis using a random effects model to estimate summary statistics for incidence (95% confidence interval) of vasa praevia (VP) based on screening cohort studies that reported a specific policy of screening for VP during the study period

Author (year)	n/N	n/1,000 pregnancies	1 per *n* pregnancies
Baulies *et al.*, 2007[26]	9/12,063	0.75 (0.34-1.42)	1,340 (706-2,933)
Rebarber *et al.*, 2013[31]	24/27,573	0.87 (0.56-1.29)	1,149 (775-1,792)
Catanzarite *et al.*, 2016[10]	96/100,481	0.96 (0.77-1.17)	1,047 (857-1,292)
Kulkarni *et al.*, 2017[34]	35/56,000	0.63 (0.44-0.87)	1,600 (1,151-2,299)
Nohuz *et al.*, 2017[35]	8/18,152	0.44 (0.19-0.87)	2,268 (1,152-5,263)
Sullivan *et al.*, 2017[11]	63/294,045	0.21 (0.17-0.27)	4,673 (3,650-6,061)
Klahr *et al.*, 2019[38]	61/37,236	1.64 (1.25-2.10)	610 (476-800)
La *et al.*, 2020[39]	19/56,045	0.34 (0.20-0.53)	2,950 (1,890-4,902)
Zhang *et al.*, 2020[12]	21/26,830	0.78 (0.49-1.20)	1,277 (836-2,062)
Gross *et al.*, 2021[40]	21/5,905	3.56 (2.20-5.43)	281 (184-455)
Sutera *et al.*, 2021[41]	24/89,600	0.27 (0.17-0.40)	3,731 (2,506-5,814)
Kamijo *et al.*, 2022[43]	14/8,723	1.60 (0.88-2.69)	625 (372-1,139)
**Pooled analysis**	**395/732,654**	**0.82 (0.53-1.18)**	**1,218 (847-1,901)**
Cochrane Q (DF)	222.18 (11)		
I^2 (inconsistency) (%) (95%CI)	95.1 (92.9-96.5)		
Egger bias (p value)	6.1508 (0.0092)		

For peer review only

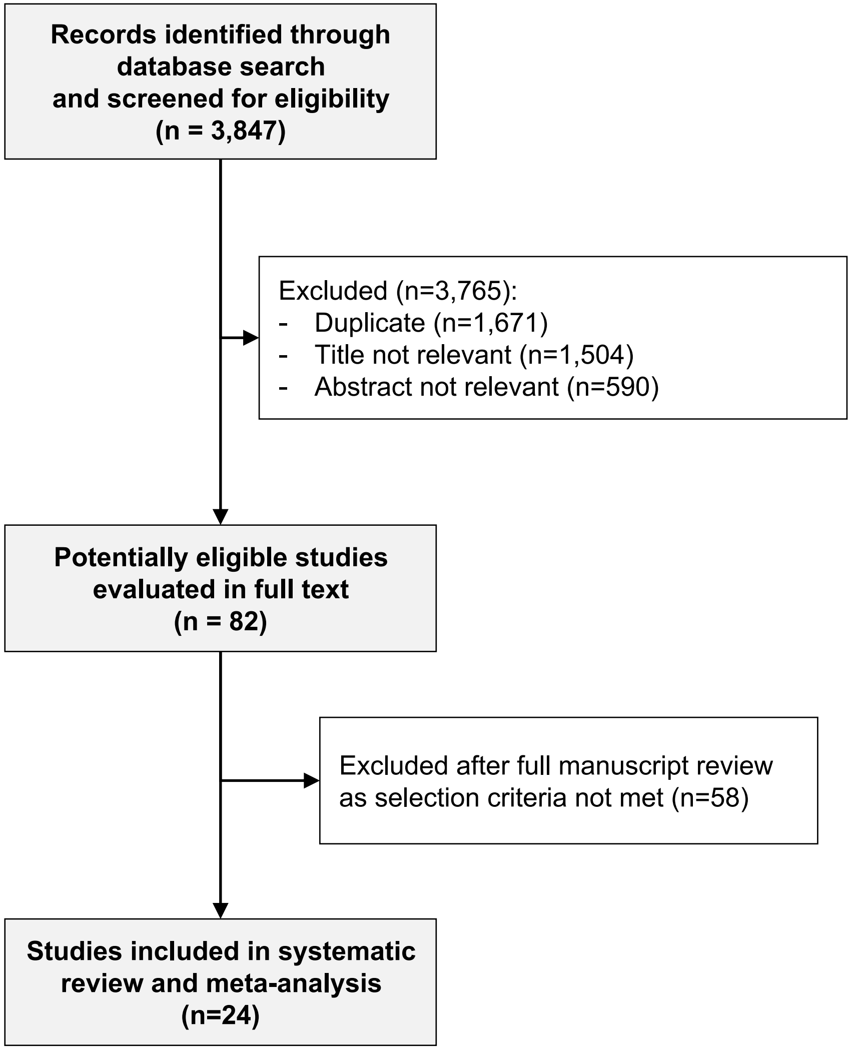
Records identified through database search and screened for eligibility (n = 3,847)
Excluded (n=3,765):
- Duplicate (n=1,671)
- Title not relevant (n=1,504)
- Abstract not relevant (n=590)
Potentially eligible studies evaluated in full text (n = 82)
Excluded after full manuscript review as selection criteria not met (n=58)
Studies included in systematic review and meta-analysis (n=24)

Figure 1.

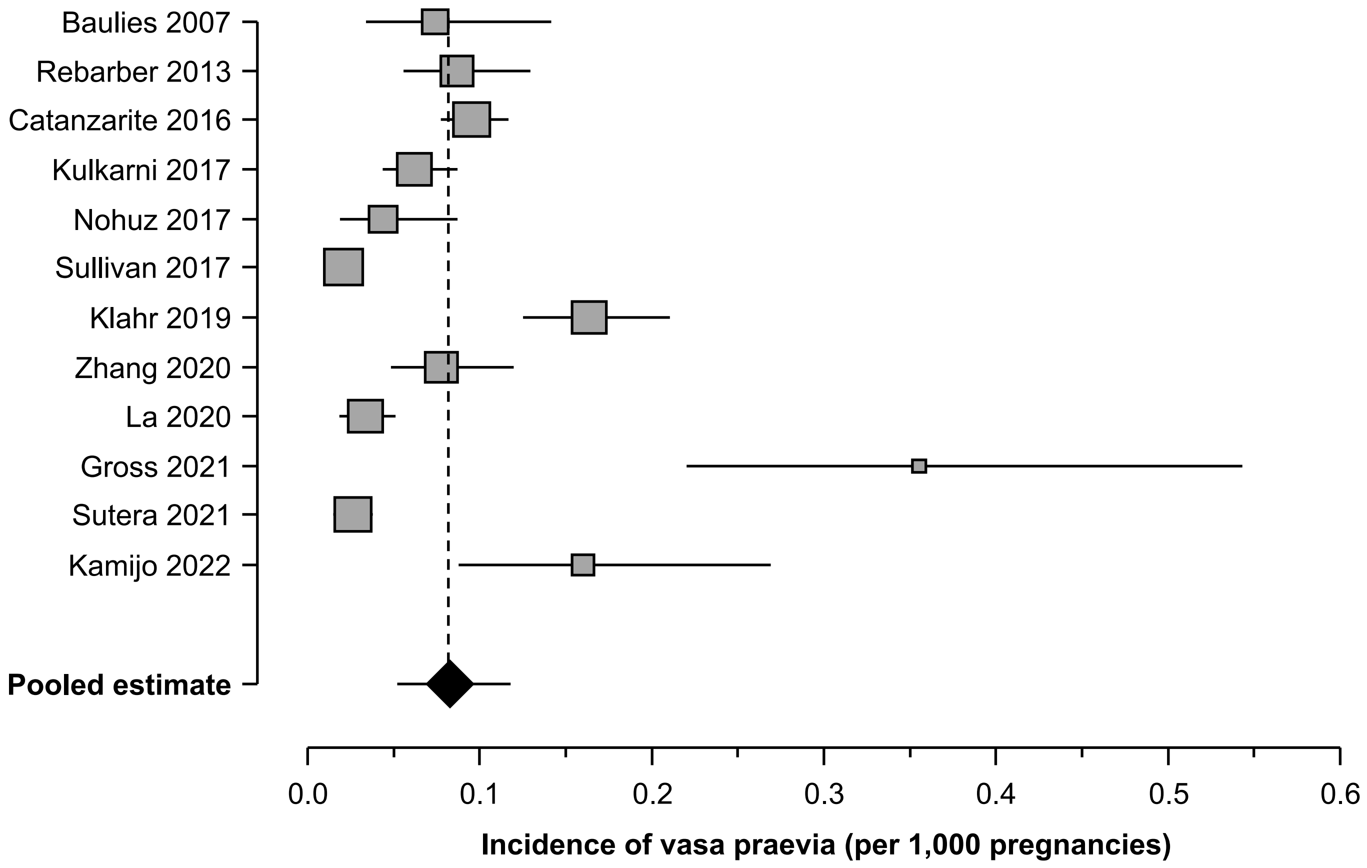
Baulies 2007
Rebarber 2013
Catanzarite 2016
Kulkarni 2017
Nohuz 2017
Sullivan 2017
Klahr 2019
Zhang 2020
La 2020
Gross 2021
Sutera 2021
Kamijo 2022
Pooled estimate
0.0
0.1
0.2
0.3
0.4
0.5
0.6
Incidence of vasa praevia (per 1,000 pregnancies)

Figure 2.

Supplementary table S1: Search Strategy for study

**MEDLINE**
1. Medline(Vasa praevia OR vasa previa).ti,ab<br>2. Medline(abnormal cord insertion).ti,ab<br>3. Medline(marginal cord insertion).ti,ab<br>4. Medline(Velamentous cord insertion).ti,ab<br>5. Medline(bilobed placenta).ti,ab<br>6. Medline(succenturiate lobe).ti,ab<br>7. Medline(accessory lobe).ti,ab<br>8. Medline*(1 OR 2 OR 3 OR 4 OR 5 OR 6 OR 7)*
**CINAHL**
1. CINAHL(Vasa praevia OR vasa previa).ti,ab<br>2. CINAHL(abnormal cord insertion).ti,ab<br>3. CINAHL(marginal cord insertion).ti,ab<br>4. CINAHL(Velamentous cord insertion).ti,ab<br>5. CINAHL(bilobed placenta).ti,ab<br>6. CINAHL(succenturiate lobe).ti,ab<br>7. CINAHL(accessory lobe).ti,ab<br>8. CINAHL*(1 OR 2 OR 3 OR 4 OR 5 OR 6 OR 7)*
**PubMed**
1. PubMed(Vasa praevia OR vasa previa).ti,ab<br>2. PubMed(abnormal cord insertion).ti,ab<br>3. PubMed(marginal cord insertion).ti,ab<br>4. PubMed(Velamentous cord insertion).ti,ab<br>5. PubMed(bilobed placenta).ti,ab<br>6. PubMed(succenturiate lobe).ti,ab<br>7. PubMed(accessory lobe).ti,ab<br>8. PubMed*(1 OR 2 OR 3 OR 4 OR 5 OR 6 OR 7)*
**EMBASE**
1. EMBASE(Vasa praevia OR vasa previa).ti,ab

For peer review only

2. EMBASE(abnormal cord insertion).ti,ab
3. EMBASE(marginal cord insertion).ti,ab
4. EMBASE(Velamentous cord insertion).ti,ab
5. EMBASE(bilobed placenta).ti,ab
6. EMBASE(succenturiate lobe).ti,ab
7. EMBASE(accessory lobe).ti,ab
8. EMBASE*(1 OR 2 OR 3 OR 4 OR 5 OR 6 OR 7)*

For peer review only

Supplementary Table 1. Methodological assessment of included studies based on Newcastle – Ottawa Score Summary

Study	Selection	Comparability	Outcome	NOS Score
Lee *et al.*, 2000[24]	★★★	★	★★★	7
Catanzarite *et al.*, 2001[8]	★★★	★	★★★	7
Schachter *et al.*, 2002[25]	★★★	★	★★★	7
Baulies *et al.*, 2007[26]	★★★	★	★★★	7
Suzuki *et al.*, 2008[27]	★★★	★	★★★	7
Smorgick *et al.*, 2010[28]	★★★	-	★★★	6
Hasegawa *et al.*, 2010[29]	★★★	★★	★★★	8
Kanda *et al.*, 2011[30]	★★★	★	★★★	7
Rebarber *et al.*, 2013[31]	★★★	★	★★★	7
Bronsteen *et al.*, 2013[32]	★★★	★	★★★	7
Hasegawa *et al.*, 2015[33]	★★★	★	★★★	7
Catanzarite *et al.*, 2016[10]	★★★	★	★★★	7
Kulkarni *et al.*, 2017[34]	★★★	★	★★★	7
Nohuz *et al.*, 2017[35]	★★★	★	★★★	7
Sullivan *et al.*, 2017[11]	★★★	★	★★★	7
Yeaton-Massey *et al.*, 2019[36]	★★	★	★★★	6
Derisbourg *et al.*, 2019[37]	★★★	★	★★★	7
Klahr *et al.*, 2019[38]	★★	★	★★★	6
La *et al.*, 2020[39]	★★★	★	★★★	7
Zhang *et al.*, 2020[12]	★★★	★	★★★	7
Gross *et al.*, 2021[40]	★★★	★	★★★	7
Sutera *et al.*, 2021[41]	★★★	★	★★★	7
Liu *et al.*, 2021[42]	★★★	★	★★★	7
Kamijo *et al.*, 2022[43]	★★★	★	★★★	7

For peer review only

Supplementary Table 2. The Preferred Reporting Items for Systematic Reviews and Meta-analysis: The PRISMA statement

Section/topic	#	Checklist item	Reported on page #
**TITLE**			
Title	1	Identify the report as a systematic review, meta-analysis, or both.	1
**ABSTRACT**			
Structured summary	2	Provide a structured summary including, as applicable: background; objectives; data sources; study eligibility criteria, participants, and interventions; study appraisal and synthesis methods; results; limitations; conclusions and implications of key findings; systematic review registration number.	2
**INTRODUCTION**			
Rationale	3	Describe the rationale for the review in the context of what is already known.	4
Objectives	4	Provide an explicit statement of questions being addressed with reference to participants, interventions, comparisons, outcomes, and study design (PICOS).	4
**METHODS**			
Protocol and registration	5	Indicate if a review protocol exists, if and where it can be accessed (e.g., Web address), and, if available, provide registration information including registration number.	5
Eligibility criteria	6	Specify study characteristics (e.g., PICOS, length of follow-up) and report characteristics (e.g., years considered, language, publication status) used as criteria for eligibility, giving rationale.	5
Information sources	7	Describe all information sources (e.g., databases with dates of coverage, contact with study authors to identify additional studies) in the search and date last searched.	5
Search	8	Present full electronic search strategy for at least one database, including any limits used, such that it could be repeated.	5 (Table S1)
Study selection	9	State the process for selecting studies (i.e., screening, eligibility, included in systematic review, and, if applicable, included in the meta-analysis).	5
Data collection process	10	Describe method of data extraction from reports (e.g., piloted forms, independently, in duplicate) and any processes for obtaining and confirming data from investigators.	5
Data items	11	List and define all variables for which data were sought (e.g., PICOS, funding sources) and any assumptions and simplifications made.	5,6

For peer review only

Risk of bias in individual studies	12	Describe methods used for assessing risk of bias of individual studies (including specification of whether this was done at the study or outcome level), and how this information is to be used in any data synthesis.	5
Summary measures	13	State the principal summary measures (e.g., risk ratio, difference in means).	5,6
Synthesis of results	14	Describe the methods of handling data and combining results of studies, if done, including measures of consistency (e.g., I^2) for each meta-analysis.	6

Section/topic	#	Checklist item	Reported on page #
Risk of bias across studies	15	Specify any assessment of risk of bias that may affect the cumulative evidence (e.g., publication bias, selective reporting within studies).	5 (Table S2/3)
Additional analyses	16	Describe methods of additional analyses (e.g., sensitivity or subgroup analyses, meta-regression), if done, indicating which were pre-specified.	6
**RESULTS**			
Study selection	17	Give numbers of studies screened, assessed for eligibility, and included in the review, with reasons for exclusions at each stage, ideally with a flow diagram.	6 (Figure 1)
Study characteristics	18	For each study, present characteristics for which data were extracted (e.g., study size, PICOS, follow-up period) and provide the citations.	6,7
Risk of bias within studies	19	Present data on risk of bias of each study and, if available, any outcome level assessment (see item 12).	7 (Table S2/3)
Results of individual studies	20	For all outcomes considered (benefits or harms), present, for each study: (a) simple summary data for each intervention group (b) effect estimates and confidence intervals, ideally with a forest plot.	6,7 (Table 1 &2)
Synthesis of results	21	Present results of each meta-analysis done, including confidence intervals and measures of consistency.	6,7
Risk of bias across studies	22	Present results of any assessment of risk of bias across studies (see Item 15).	6,7
Additional analysis	23	Give results of additional analyses, if done (e.g., sensitivity or subgroup analyses, meta-regression [see Item 16]).	7
**DISCUSSION**			

For peer review only

Summary of evidence	24	Summarize the main findings including the strength of evidence for each main outcome; consider their relevance to key groups (e.g., healthcare providers, users, and policy makers).	8
Limitations	25	Discuss limitations at study and outcome level (e.g., risk of bias), and at review-level (e.g., incomplete retrieval of identified research, reporting bias).	9
Conclusions	26	Provide a general interpretation of the results in the context of other evidence, and implications for future research.	8,9
**FUNDING**			
Funding	27	Describe sources of funding for the systematic review and other support (e.g., supply of data); role of funders for the systematic review.	11

For peer review only

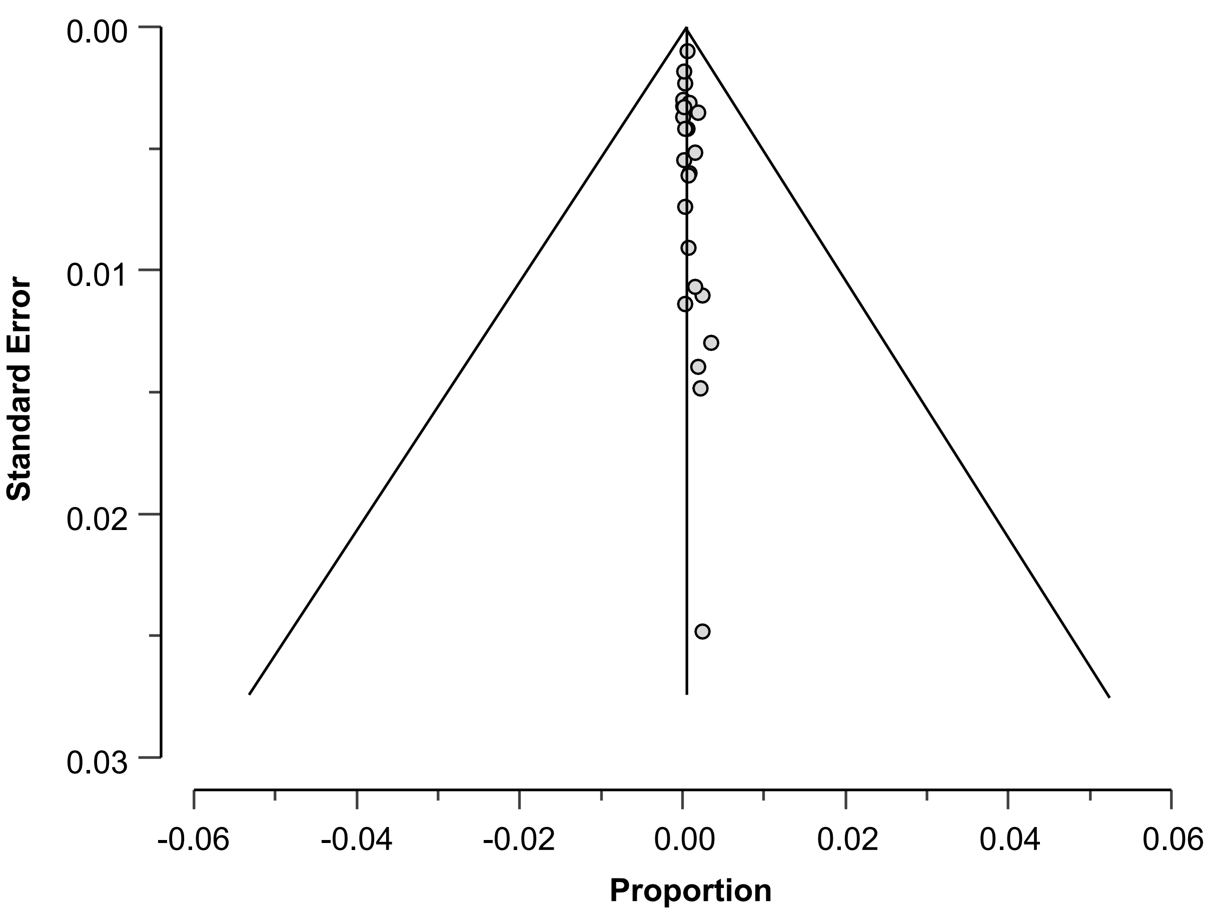

Supplementary Figure F1.

BMJ Open

Incidence of Vasa praevia: - A systematic review and meta-analysis

Journal:	*BMJ Open*
Manuscript ID	bmjopen-2023-075245.R1
Article Type:	Original research
Date Submitted by the Author:	08-Aug-2023
Complete List of Authors:	Zhang, Weiyu; Medway NHS Foundation Trust, Medway Fetal and Maternal Medicine Centre<br>Giacchino, Tara; Medway NHS Foundation Trust, Medway Fetal and Maternal Medicine Centre<br>Chanyarungrojn, Pannapat Amy; Medway NHS Foundation Trust, Medway Fetal and Maternal Medicine Centre<br>Ionescu, Olivia; Medway NHS Foundation Trust, Medway Fetal and Maternal Medicine Centre<br>Akolekar, Ranjit; Canterbury Christ Church University, Institute of Medical Sciences; Medway NHS Foundation Trust, Medway Fetal and Maternal Medicine Centre
**Primary Subject Heading**:	Obstetrics and gynaecology
Secondary Subject Heading:	Evidence based practice
Keywords:	Prenatal diagnosis < OBSTETRICS, Fetal medicine < OBSTETRICS, Systematic Review

SCHOLARONE™
Manuscripts

I, the Submitting Author has the right to grant and does grant on behalf of all authors of the Work (as defined in the below author licence), an exclusive licence and/or a non-exclusive licence for contributions from authors who are: i) UK Crown employees; ii) where BMJ has agreed a CC-BY licence shall apply, and/or iii) in accordance with the terms applicable for US Federal Government officers or employees acting as part of their official duties; on a worldwide, perpetual, irrevocable, royalty-free basis to BMJ Publishing Group Ltd ("BMJ") its licensees and where the relevant Journal is co-owned by BMJ to the co-owners of the Journal, to publish the Work in this journal and any other BMJ products and to exploit all rights, as set out in our licence.

The Submitting Author accepts and understands that any supply made under these terms is made by BMJ to the Submitting Author unless you are acting as an employee on behalf of your employer or a postgraduate student of an affiliated institution which is paying any applicable article publishing charge ("APC") for Open Access articles. Where the Submitting Author wishes to make the Work available on an Open Access basis (and intends to pay the relevant APC), the terms of reuse of such Open Access shall be governed by a Creative Commons licence – details of these licences and which Creative Commons licence will apply to this Work are set out in our licence referred to above.

Other than as permitted in any relevant BMJ Author's Self Archiving Policies, I confirm this Work has not been accepted for publication elsewhere, is not being considered for publication elsewhere and does not duplicate material already published. I confirm all authors consent to publication of this Work and authorise the granting of this licence.

For peer review only

Title: Incidence of Vasa praevia: - A systematic review and meta-analysis

Authors: Weiyu Zhang[1], Tara Giacchino[1], Pannapat Amy Chanyarungrojn[1], Olivia Ionescu[1], Ranjit Akolekar[1,2]

Key words: Vasa praevia, Incidence, Stillbirths, Prenatal diagnosis

Short Title: Incidence of vasa praevia

Affiliation:

1. Medway Fetal and Maternal Medicine Centre, Medway Maritime Hospital, United Kingdom
2. Institute of Medical Sciences, Canterbury Christ Church University, Rowan William's Court, Kent, United Kingdom

Corresponding author:

Professor R Akolekar, MD, MRCOG
Institute of Medical Sciences,
Canterbury Christ Church University,
Rowan William's Court,
Chatham, Kent ME4 4UF
Email: ranjit.akolekar@canterbury.ac.uk

Word count: 3,144

For peer review only

For peer review only - http://bmjopen.bmj.com/site/about/guidelines.xhtml

ABSTRACT

Objectives: To derive accurate estimates of the incidence of vasa praevia (VP) in a routine population of unselected pregnancies

Design: Systematic review and meta-analysis

Data sources: A search of MEDLINE, EMBASE, CINHAL and the Cochrane database was performed to review relevant citations reporting outcomes in pregnancies with VP from January 2000 till 5th April 2023

Eligibility criteria for selection of studies: Prospective or retrospective cohort or population studies that provided data regarding VP cases in routine unselected pregnancies during the study period. We included studies published in the English language after the year 2000 to reflect contemporary obstetric and neonatal practice.

Data extraction and synthesis: Two reviewers independently screened the retrieved citations and extracted data. The methodological quality of studies was assessed using the Newcastle-Ottawa Scale (NOS) and quality of systematic review was validated with PRISMA (Preferred Reporting Items for Systematic reviews and Meta-Analyses).

Results: A total of 3,847 citations were screened and 82 full-text manuscripts were retrieved for analysis. There were 24 studies that met inclusion criteria, of which 12 studies reported prenatal diagnosis with a systematic protocol of screening. There were 1,320 pregnancies with VP in a total population of 2,278,561 pregnancies; the weighted pooled incidence of VP was 0.79 (95%CI: 0.59-1.01) per 1,000 pregnancies, corresponding to 1 case of VP per 1,271 (95% CI: 990-1,692) pregnancies. Nested sub-analysis of studies reporting screening for VP based on a specific protocol identified 395 pregnancies with VP in a population of 732,654 pregnancies with weighted pooled incidence of 0.82 (95%CI: 0.53-1.18) per 1,000 pregnancies (1 case of VP per 1,218 (95% CI: 847-1,901) pregnancies.

Conclusion: The incidence of VP in unselected pregnancies is 1 in 1,218 pregnancies. This is higher than is previously reported and can be used as a basis to assess whether screening for this condition should be part of routine clinical practice. Incorporation of strategies to screen for VP in routine clinical practice is likely to prevent 5-6% of stillbirths.

PROSPERO registration number: CRD42020125495.

For peer review only - http://bmjopen.bmj.com/site/about/guidelines.xhtml

STRENGTHS AND LIMITATIONS OF THE STUDY

- Our study provides an accurate estimate of incidence of vasa praevia based on a systematic review of studies with an *a priori* designed protocol registered on international register of systematic reviews (PROSPERO).
- The study only includes cohort studies providing data regarding vasa praevia and control pregnancies delivered during the study period.
- We only included studies that were published since the year 2000 to reflect contemporary clinical practice.
- We undertook a sub-group nested analysis in studies that reported a systematic and specific policy of screening for vasa praevia in their hospital and reported both cases of vasa praevia as well as the population of control pregnancies.
- A limitation of our study is that we did not provide separate incidence for singleton and multiple pregnancies.

For peer review only

INTRODUCTION

Vasa praevia (VP) is defined as presence of fetal blood vessels, arterial or venous, unsupported by placental tissue or umbilical cord traversing the amniotic membranes in the lower uterine segment in close proximity to the internal cervical os, [1-5]. These unsupported fetal vessels can be damaged either during the antenatal period or in labour leading to severe hypovolemic shock and haemorrhagic fetal death, [4,6,7]. There is considerable evidence suggesting that prenatal diagnosis of VP improves perinatal survival and lack of antenatal detection is associated with a high-risk of stillbirths, neonatal deaths and an increased risk of hypoxic morbidity in the survivors, [8-13]. Despite the compelling evidence in favour of prenatal diagnosis in improving pregnancy outcomes in pregnancies with VP, there are currently no recommendations for screening for VP in routine clinical practice, [14]. The United Kingdom (UK) National Screening Committee examined the evidence with regard to screening for VP and reported that there was low volume of evidence on the epidemiology of VP or its outcomes in the UK to justify screening, [15].

A systematic review examined the incidence of VP and reported that there was a huge variation in studies reporting VP incidence ranging from 0.19-2.21 per 1,000 pregnancies (1 per 452 to 1 per 5,882 pregnancies) with a mean estimated incidence of 0.6 per 1,000 pregnancies (1 per 1,666 pregnancies), [16]. This systematic review had significant heterogeneity and variation as it included not just cohort studies but case-control studies and those from high-risk populations. The estimated incidence in this systematic review is likely to have been underestimated as many of the included studies reported data from 80s and 90s when VP was more likely to be an incidental rather than an intentional diagnosis. In a recent large prospective observational screening study of more than 26,000 pregnancies, we demonstrated the impact of routine screening for VP based on a 2-stage screening strategy and reported that the incidence of VP was about 1 in 1,277 pregnancies, [12]. The protocol states that stage 1 includes checking for umbilical cord insertion and placental location; those that are velamentous cord insertions in lower segment and those with a low-lying placenta are classed as at-risk and stage 2 would include a transvaginal assessment with colour Doppler ultrasound to check for presence of any fetal vessels in the vicinity of the cervix for a diagnosis of VP, [12].

The objective of this study was to undertake a systematic review of literature and undertake a meta-analysis to estimate an accurate incidence of VP in a routine population of unselected pregnancies based on analysis of studies that reported a policy of routinely screening for VP.

For peer review only - http://bmjopen.bmj.com/site/about/guidelines.xhtml

METHODS

Data sources and search strategy

This systematic review and meta-analysis was undertaken based on an *a-priori* designed study protocol recommended for systematic reviews and meta-analyses, [17]. The study protocol for the systematic review was registered in advance with the PROSPERO International prospective register of systematic reviews (Registration number: CRD42020125495) as a study to investigate perinatal outcomes as well as incidence of VP. An electronic search of MEDLINE, EMBASE and The Cochrane Library was carried out on 30th October 2022 and repeated on 5th April 2023 utilising combinations of the relevant Medical Subject Heading (MeSH) terms, key words and word variants for “vasa praevia”, “abnormal cord insertion”, “velamentous cord”, marginal cord”, “bilobed placenta” and “succenturiate lobe”. The search and selection criteria were restricted to studies reported in the English language and are outlined in Supplementary table S1.

Selection criteria for studies and data extraction

The studies eligible for inclusion in this study were prospective or retrospective cohort studies and population-based studies reporting cases with VP in which the authors reported data about the total screened population during the study period. We only included studies published after 2000 to reflect contemporary clinical practice. We did a nested sub-analysis for studies which reported in their methodology that they had a routine screening policy for assessing umbilical cord insertion and VP. The citations were examined by two reviewers (WZ and PC) to produce a list of relevant studies after exclusion of duplicates, those that did not fit selection criteria after review of title and abstract, those that were case-reports, letter to editors, review articles or conference abstracts and those that were case series or case-control studies. The reference lists of relevant articles were searched, and any inconsistencies were resolved by discussion with an internal third reviewer (RA).

The citations retrieved following this search strategy were examined for relevance to this study to investigate incidence based on above eligibility criteria and in each study, we extracted information about authors, study location, years of enrolment for cases and controls, study design, sample size, whether the study was single or multi-centre, whether the study was in high-risk population or unselected general pregnant population, and whether there was a routine policy of screening for umbilical cord insertion and VP during the study period in the reported studies. Data extracted for each study was inputted into contingency tables.

For peer review only - http://bmjopen.bmj.com/site/about/guidelines.xhtml

Quality Assessment

The methodological quality of studies included in the review was assessed using the Newcastle-Ottawa Scale (NOS), [18]. We used PRISMA (Preferred Reporting Items for Systematic reviews and Meta-Analyses) to ensure standardised reporting of studies in our systematic review. The PRISMA statement for this study included a checklist and a flow chart to allow uniform and transparent reporting of the systematic review and meta-analysis, [19].

Estimation of summary statistics for incidence of vasa praevia (VP)

Data were entered in contingency tables for cases and control pregnancies to calculate weighted pooled incidence (95% confidence intervals [CI]). The incidence of VP was reported as total number of cases per 1,000 pregnancies as well as total number of pregnancies for every 1 case of VP. Summary statistics (95%CI) from each study were then combined to obtain a pooled estimate which was calculated as a weighted average of the individual study estimates. The pooled summary statistics were estimated using random-effects model (REM) to allow for assessment of between-study variability in results by weighting studies using a combination of their own variance as well as the between-study variance, [20]. The heterogeneity between studies was estimated using Cochrane's heterogeneity statistic Q, and the I^2 statistic. Regression analysis was undertaken to examine factors providing a significant contribution to the prediction of incidence of VP. We also estimated the potential impact of introducing a policy for screening for VP in preventing stillbirths in the UK based on annual number of total births and stillbirths reported by the Office of National Statistics, National Records of Scotland and Northern Ireland Statistics and Research Agency, [21-23]. The statistical software package StatsDirect version 2.7.9 (StatsDirect Ltd, Cheshire, United Kingdom) and MedCalc Statistical Software version 16.4.3 (MedCalc Software, Ostend, Belgium) were used for data analysis.

Patient and public involvement

No patients were involved in setting the research question, in developing plans for design, interpretation, reporting or implementation of the study.

RESULTS

Data search results

The electronic search from the databases yielded 3,847 potential citations; of these, we excluded 1,671 citations as they were duplicates, 1,504 after review of study title and 590 citations after review of the abstract. We retrieved 82 manuscripts in full text for detailed

For peer review only - http://bmjopen.bmj.com/site/about/guidelines.xhtml

assessment and excluded a further 58 studies that did not meet the selection criteria, thus finally including 24 studies for systematic review and meta-analysis. The search criteria are outlined in supplementary table S1, and the study selection process is outlined in the flow chart in Figure 1.

Characteristics of studies included in the systematic review

There were 24 studies included in the systematic review, [8,10-12,24-43], which provided data regarding incidence of VP. There were 7 cohort studies published in the first decade (2000 to 2010), [8,24-29] and 17 studies in the second decade (2011 to 2020), [10-12,30-43]. The published studies were from across different geographical areas; 8 studies from United states, [8,10,24,31,32,34,36,38] 5 studies from Japan, [27,29,30,33,43], 6 from Europe, [12,26,35,37,40,41], 2 each from Australia, [11,39] and Israel, [25,28] and 1 from China, [42]. All 24 studies reported the total screened population during the study period and the number of cases of VP detected, thus providing a basis of estimating incidence of VP. Screening studies which report a policy-based assessment of an unselected cohort of pregnancies are more likely to reflect the true incidence of VP, compared to studies that included an incidental diagnosis of VP. Therefore, we identified studies that described a specific protocol for assessment of all pregnancies during the study period for prenatal diagnosis of VP after excluding those that had a variable protocol for identification of at-risk pregnancies, those that were carried out in a specific high-risk population such as those with velamentous cord insertion, [37], succenturiate lobe, [27], *in vitro* fertilisation pregnancies, [25], or those who were only delivered by caesarean section, [36]. After exclusion of above studies, we identified 12 studies, [10-12,26,31,34,35,38-41,43], in which the authors reported that they had a systematic protocol to document the placental location and umbilical cord insertion at the first or second trimester scan; those that were suspected to have VP were further scanned with transvaginal ultrasound to confirm the diagnosis. In the 12 screening protocol-based studies included, the authors reported confirmation of the presence of VP postnatally (Supplementary table S4).

Assessment of quality and heterogeneity of studies

The methodological quality of studies included in this systematic review was assessed using the NOS. The rating of the included studies according to the NOS based on selection, comparability and outcome are shown in Supplementary table S2. The PRISMA guidance was followed for reporting this meta-analysis and is reported in Supplementary table S3). The funnel plot of included studies demonstrating the publication bias is shown in Supplementary figure F1.

For peer review only - http://bmjopen.bmj.com/site/about/guidelines.xhtml

Incidence of vasa praevia (VP)

There were 24 studies that reported data about cases with VP with a corresponding cohort of total number of unselected pregnancies screened in a routine population during the study period, [8,10-12,24-43]. There were 1,320 pregnancies with VP in a total population of 2,278,561 pregnancies. The weighted pooled incidence of VP was 0.79 (95%CI: 0.59-1.01) per 1,000 pregnancies screened, which corresponded to an incidence of 1 case of VP per 1,271 (95% CI: 990-1,692) pregnancies (Table 1).

There were 12 studies that reported data regarding incidence of VP along with the total pregnancies screened based on a specific screening protocol, [10-12,26,31,34,35,38-41,43]. There were 395 pregnancies with VP in a total population of 732,654 pregnancies. The weighted pooled incidence of VP was 0.82 (95%CI: 0.53-1.18) per 1,000 pregnancies screened, which corresponded to an incidence of 1 case of VP per 1,218 (95% CI: 847-1,901) pregnancies (Table 2, Figure 2).

Regression analysis demonstrated that in prediction of the incidence of VP, there was a significant independent contribution from the publication date of the study, whether the study was published in the first decade (2000-2010) or in the second decade (2011-2022) ($p<0.011$) but there was no significant association with the country in which the study was conducted ($p=0.408$) ($R^2=0.208$; $p<0.05$). There was a significant increase in incidence of VP from studies published in the second decade (2011-2022) compared to first decade (2000-2010) (OR: 2.60; 95%CI: 2.08-3.25) ($p<0.01$).

Potential impact on prevention of stillbirths in United Kingdom

There were total of 697,551 births in the UK with 2,866 (0.41%) stillbirths in 2021. Given an incidence of VP of 1 in 1,218 pregnancies, there would be an estimated 573 pregnancies with VP per year in the UK. Based on estimates from the systematic review and meta-analysis reporting perinatal survival in pregnancies with VP, the livebirth rate in pregnancies with VP with and without prenatal diagnosis is 98.6% (95%CI: 96.7-99.7) and 72.1% (95%CI: 50.6-89.4), respectively; therefore, the expected number of stillbirths in pregnancies with VP in those with and without prenatal diagnosis would be 8 (95%CI: 2-19) and 160 (95% CI: 61-283). Therefore, the potential impact of introducing a policy of screening for VP in clinical practice could contribute to prevention of 5.58% (95%CI: 2.13-9.87) of stillbirths in the UK. This is based on the assumption that VP is causal to the fetal death but in some pregnancies with VP, other risk factors and / or pregnancy complications may also play a role.

For peer review only - http://bmjopen.bmj.com/site/about/guidelines.xhtml

DISCUSSION

Principal findings of the study

The findings of our systematic review and meta-analysis demonstrate that the incidence of VP in an unselected population of pregnancies is 1 in 1,218 pregnancies. The estimated incidence of VP in our study is based on weighted pooled estimates calculated firstly, from meta-analysis of studies published in the last 20 years to reflect contemporary obstetric and neonatal practice and secondly, by inclusion of those studies that reported a specific policy of assessment of placental location and umbilical cord insertion for prenatal diagnosis of VP in their routine practice. The incidence of VP was not affected by the study location or where the study was carried out, reflecting that in centres that have a specific protocol for assessment of VP as described above, there is no significant variation in reported incidence. Our study demonstrates that if screening for prenatal diagnosis of VP was introduced in routine clinical practice, it can potentially prevent 5-6% of stillbirths.

Comparison with previous studies

The results of our study demonstrate that VP is not an uncommon obstetric complication and has an incidence of 1 in 1,218 pregnancies, which is higher than that reported in previous studies, [16]. A previous systematic review reported a wide variation in the incidence of VP from 1 per 452 to 5,882 pregnancies with a mean incidence of 1 in 1,666 pregnancies. The authors included a wide range of studies including case-control studies, retrospective cohort studies, studies carried out on high-risk populations (such as those with pregnancies conceived with *in vitro* fertilisation, studies examining outcomes in pregnancies with bilobed placenta or in those that had a velamentous cord insertion), those that reported an incidental diagnosis of VP rather than cases detected in an unselected population based on a systematic protocol of screening and inclusion of studies that did not report whether there was postnatal confirmation of prenatal findings of VP, thus leading to a wide heterogeneity in the results and potentially underestimation of the incidence. Moreover, the authors included studies reporting data from 80s and 90s, which are more likely to be incidentally diagnosed cases of VP rather than as a result of intentional screening.

Implications for clinical practice

Our study highlights that the incidence of VP is much higher than is previously reported. The results of our study provide an accurate estimate of the incidence which can for the basis for further studies to investigate whether VP screening should be incorporated in routine practice as not only it is more common than previously believed, but more importantly, prenatal diagnosis can prevent adverse outcomes associated with VP. There is robust and unequivocal

For peer review only - http://bmjopen.bmj.com/site/about/guidelines.xhtml

evidence from several studies, including our cohort study, suggesting that prenatal diagnosis of VP is associated with high rates of livebirths and perinatal survival, [10-12,30,34,35,40-42]. A recent systematic review and meta-analysis by our group summarised and confirmed the results from previous studies demonstrating that prenatal diagnosis of VP is associated with a 99% rate of perinatal survival but a lack of an antenatal diagnosis increases the risk of a perinatal hypoxic morbidity 50-fold and that of perinatal death by 25-fold, [13]. There are few other examples in obstetric practice which demonstrate such a significant and stark impact of prenatal diagnosis on improving pregnancy outcomes such as the prenatal diagnosis of VP. There are 12 relatively recent studies, [10-12,26,31,34,35,38-41,43], which report that systematic and routine screening for VP is feasible in routine clinical practice; in fact, our recent prospective observational study of more than 26,000 pregnancies demonstrates that a 2-stage screening strategy for VP can be easily implemented in routine clinical practice and such a policy is associated with a high rate of prenatal diagnosis and good perinatal outcomes, [12]. The recommendations for screening for VP in routine practice is hindered by evidence about accurate incidence in routine pregnant population. Our study should pave the way for further studies that investigate strategies of introduction screening for VP in routine pregnancies.

<u>Strengths and limitations</u>

The strength of our study is that it summarises results of all studies reported in the last two decades and provides accurate summary estimates for incidence of VP in current obstetric practice. We used a standardised methodology for conducting this systematic review and meta-analysis with design of an *a-priori* protocol, registration with the international register at PROSPERO, a comprehensive search strategy, appropriate quality assessment of studies included in systematic review with NOS and standardised reporting of systematic review with PRISMA checklist. The limitations of our study relate to standard biases associated with meta-analysis such as inclusion of studies with different sample size, methodology and design which may introduce heterogeneity in the analysis. However, as a means to minimize the effects of such variation and heterogeneity, we reviewed all these studies in detail to select and identify those studies that provide data from low-risk unselected pregnancies rather than high-risk populations. This allowed us to do a nested meta-analysis to estimate an accurate weighted pooled summary statistic, which is more reflective of true population incidence rather than just a pooled estimated from all reported studies. We used a random effects model to mitigate the impact of heterogeneity as this model allows for assessment of between-study variability in results by weighing studies using a combination of their own variance as well as the between-study variance. Furthermore, we performed regression analyses to examine temporal and spatial trends in VP incidence such as the publication dates as well as the location of where the studies were carried out. A confounding factor and a limitation of our study is that we did

For peer review only

not exclude multiple pregnancies and this incidence is the total incidence in singleton and multiple pregnancies; the incidence in multiple pregnancies could potentially be higher but requires further studies to investigate this.

Conclusions

VP is not an uncommon obstetric complication. The incidence of VP in an unselected population is 1 in 1,218 pregnancies, which is considerably higher than previously reported estimates. Further research studies should be undertaken to model and investigate strategies for incorporating prenatal screening for VP in routine clinical practice, given the feasibility of screening and major impact on prevention of stillbirths and hypoxic morbidity with prenatal diagnosis of VP.

Acknowledgement: We are grateful to the Medway Library and Knowledge Service for their help and assistance with search strategy. This study is a part of the PhD thesis of Weiyu Zhang for the Canterbury Christ Church University.

Author statement: WZ, PC, TG and OI contributed to literature search, study selection, data extraction and assessment of study quality. WZ and RA wrote the initial and final draft of the manuscript. RA conceived the study and supervised the data extraction, discussed studies for inclusion following initial review and agreed the final draft of the manuscript for submission. All authors reviewed the initial and final draft of the manuscript and contributed to the intellectual content.

Data availability statement: Data are available upon reasonable request.

Ethics approval: No ethical approval was required for this study.

Conflict of interest: None

Funding: No funding was received for this study.

For peer review only - http://bmjopen.bmj.com/site/about/guidelines.xhtml

REFERENCES

1. McNair AJ. Placenta praevia, with vasa praevia; Caesarean section. *Proc R Soc Med* 1921; 14: 195-196.
2. Alment EA. Vasa praevia simulating antepartum hemorrhage. *Br Med J* 1949; 2: 1273.
3. Sinkey RG, Odibo AO, Dashe JS. Diagnosis and management of vasa previa. Society of Maternal-Fetal (SMFM) Publications Committee. *Am J Obstet Gynecol* 2015; 213: 615-619.
4. Oyelese KO, Turner M, Lees C, Campbell S. Vasa previa: an avoidable obstetric tragedy. *Obstet Gynecol Surv* 1999; 54: 138–45.
5. Catanzarite V, Oyelese Y. Diagnosis and management of vasa previa. *Am J Obstet Gynecol* 2016; 214: 764.
6. Robert JA, Sepulveda W. Fetal exsanguination from ruptured vasa previa: still a catastrophic event in modern obstetrics. *J Obstet Gynaecol* 2003; 23: 574.
7. Antoine C, Young BK, Silverman F, Greco MA,lvarez SP. Sinusoidal fetal heart rate pattern with vasa previa in twin pregnancy. *J Reprod Med* 1982; 27: 295–300.
8. Catanzarite V, Maida C, Thomas W, Mendoza A, Stanco L, Piacquadio KM. Prenatal sonographic diagnosis of vasa previa: ultrasound findings and obstetric outcome in ten cases. *Ultrasound Obstet Gynecol* 2001; 18:109-115.
9. Oyelese Y, Catanzarite V, Prefumo F, Lashley S, Schachter M, Tovbin Y, Goldstein V, Smulian JC. Vasa previa: the impact of prenatal diagnosis on outcomes. *Obstet Gynecol* 2004; 103: 937-942.
10. Catanzarite V, Cousins L, Daneshmand S, Schwendemann W, Casele H, Adamczak J, Tith T, Patel A. Prenatally Diagnosed Vasa Previa: A Single-Institution Series of 96 Cases. *Obstet Gynecol* 2016; 128: 1153-1161.
11. Sullivan EA, Javid N, Duncombe G, Li Z, Safi N, Cincotta R, Homer CSE, Halliday L, Oyelese Y. Vasa Previa Diagnosis, Clinical Practice, and Outcomes in Australia. *Obstet Gynecol* 2017; 130: 591-598.
12. Zhang W, Geris S, Beta J, Ramadan G, Nicolaides KH, Akolekar R. Prevention of stillbirth: impact of two-stage screening for vasa previa. *Ultrasound Obstet Gynecol*. 2020; 55: 605-612.
13. Zhang W, Geris S, Al-Emara N, Ramadan G, Sotiriadis A, Akolekar R. Perinatal survival in pregnancies with a prenatal diagnosis of vasa praevia: a systematic review and meta-analysis. *Ultrasound Obstet Gynecol*. 2021; 57: 710-719.
14. Jauniaux E, Alfirevic Z, Bhide AG, Burton GJ, Collins SL, Silver R; Royal College of Obstetricians and Gynaecologists. Vasa Praevia: Diagnosis and Management: Green-top Guideline No. 27b. *BJOG*. 2019; 126: e49-e61.

For peer review only - http://bmjopen.bmj.com/site/about/guidelines.xhtml

15. Screening for Vasa praevia: An evidence map to outline the volume and type of evidence related to screening for vasa praevia for the UK National Screening Committee: Version 1.1: Costello Medical: UK National Screening Committee. March 2023. Vasa praevia - UK National Screening Committee (UK NSC) - GOV.UK (view-health-screening-recommendations.service.gov.uk). Accessed 28th April 2023.
16. Ruiter L, Kok N, Limpens J, Derks JB, de Graaf IM, Mol B, Pajkrt E. Incidence of and risk indicators for vasa praevia: a systematic review. *BJOG.* 2016; 23: 1278-87.
17. NHS Centre for Reviews and Dissemination. Systematic reviews: CRD's guidance for undertaking reviews in health care. University of York: York, UK, 2009.
18. Wells GA, Shea B, O'Connell D, Peterson J, Welch V, Losos M, Tugwell P. The Newcastle-Ottawa Scale (NOS) for assessing the quality of nonrandomized studies in meta-analysis. http://www.ohri.ca/programs/clinical_epidemiology/oxford.asp.
19. Moher D, Liberati A, Tetzlaff J, Altman DG, The PRISMA Group. Preferred Reporting Items for Systematic Reviews and Meta-Analyses: The PRISMA Statement. *BMJ* 2009; 39: b2535.
20. DerSimonian R, Laird N: Meta-analysis in clinical trials. *Control Clin Trials* 1986; 7: 177-188.
21. Births in England and Wales: Summary tables: August 2022. https://www.ons.gov.uk/peoplepopulationandcommunity/birthsdeathsandmarriages/livebirths/bulletins/birthsummarytablesenglandandwales/2021. Accessed 28th April 2023.
22. Vital events reference tables. National records of Scotland. June 2022. https://www.nrscotland.gov.uk/statistics-and-data/statistics/statistics-by-theme/vital-events/general-publications/vital-events-reference-tables/2021. Accessed 28th April 2023.
23. Stillbirths: Northern Ireland Statistics and Research Agency. November 2022. https://www.nisra.gov.uk/publications/stillbirth-and-infant-death-numbers. Accessed 28th April 2023.
24. Lee W, Lee VL, Kirk JS, Sloan CT, Smith RS, Comstock CH. Vasa previa: prenatal diagnosis, natural evolution, and clinical outcome. *Obstet Gynecol* 2000; 95: 572-576.
25. Schachter M, Tovbin Y, Arieli S, Friedler S, Ron-El R, Sherman D. In vitro fertilisation is a risk factor for vasa praevia. *Fertil Steril*. 2002; 78:642-643.
26. Baulies S, Maiz N, Muñoz A, Torrents M, Echevarría M, Serra B. Prenatal ultrasound diagnosis of vasa praevia and analysis of risk factors. *Prenat Diagn* 2007; 27: 595-599.
27. Suzuki S, Igarashi M. Clinical significance of pregnancies with succenturiate lobes of placenta. *Arch Gynecol Obstet*. 2008; 277:299-301.

For peer review only - http://bmjopen.bmj.com/site/about/guidelines.xhtml

28. Smorgick N, Tovbin Y, Ushakov F, Vaknin Z, Barzilay B, Herman A, Maymon R Is neonatal risk from vasa previa preventable? The 20-year experience from a single medical center. *J Clin Ultrasound* 2010; 38: 118-22.

29. Hasegawa J, Farina A, Nakamura M, Matsuoka R, Ichizuka K, Sekizawa A, Okai T. Analysis of the ultrasonographic findings predictive of vasa previa. *Prenat Diagn*. 2010; 30: 1121-1125.

30. Kanda E, Matsuda Y, Kamitomo M, Maeda T, Mihara K, Hatae M. Prenatal diagnosis and management of vasa previa: a 6-year review. *J Obstet Gynaecol Res* 2011; 37: 1391-1396.

31. Rebarber A, Dolin C, Fox NS, Klauser CK, Saltzman DH, Roman AS. Natural history of vasa previa across gestation using a screening protocol. *J Ultrasound Med* 2014; 33: 141-147.

32. Bronsteen R, Whitten A, Balasubramanian M, Lee W, Lorenz R, Redman M, Goncalves L, Seubert D, Bauer S, Comstock C. Vasa previa: clinical presentations, outcomes, and implications for management. *Obstet Gynecol*. 2013; 122: 352-357.

33. Hasegawa J, Arakaki T, Ichizuka K, Sekizawa A. Management of vasa previa during pregnancy. *J Perinat Med*. 2015; 43: 783-784.

34. Kulkarni A, Powel J, Aziz M, Shah L, Lashley S, Benito C, Oyelese Y. Vasa Previa: prenatal diagnosis and outcomes: thirty-five cases from a single maternal-fetal medicine practice. *J Ultrasound Med* 2018; 37: 1017-1024.

35. Nohuz E, Boulay E, Gallot D, Lemery D, Vendittelli F. Can we perform a prenatal diagnosis of vasa previa to improve its obstetrical and neonatal outcomes? *J Gynecol Obstet Hum Reprod*. 2017; 46: 373-377.

36. Yeaton-Massey A, Girsen AI, Mayo JA, Blumenfeld YJ, El-Sayed YY, Stevenson DK, Shaw GM. Vasa previa and extreme prematurity: a population-based study. *J Perinatol. 2019;* 39:475-480.

37. Derisbourg S, Boulay A, Lamy C, Barlow P, Van Rysselberge M, Thomas D, Rozenberg S, Daelemans C. First trimester ultrasound prediction of velamentous cord insertions: a prospective study. *J Matern Fetal Neonatal Med. 2021*; 34:2642-2648.

38. Klahr R, Fox NS, Zafman K, Hill MB, Connolly CT, Rebarber A. Frequency of spontaneous resolution of vasa previa with advancing gestational age. *Am J Obstet Gynecol. 2019*; 221:646.e1-646.e7.

39. La S, Melov SJ, Nayyar R. Are we over-diagnosing vasa praevia? The experience and lessons learned in a tertiary centre. *Aust N Z J Obstet Gynaecol. 2021*; 61:217-222.

40. Gross A, Markota Ajd B, Specht C, Scheier M. Systematic screening for vasa previa at the 20-week anomaly scan. *Acta Obstet Gynecol Scand. 2021;* 100:1694-1699.

For peer review only

For peer review only - http://bmjopen.bmj.com/site/about/guidelines.xhtml

466 41. Sutera M, Garofalo A, Pilloni E, Parisi S, Alemanno MG, Menato G, Sciarrone A, Viora E. Vasa previa: when antenatal diagnosis can change fetal prognosis. *J Perinat Med. 2021*; 49: 915-922.

469 42. Liu N, Hu Q, Liao H, Wang X, Yu H. Vasa previa: Perinatal outcomes in singleton and multiple pregnancies. *Biosci Trends. 2021*; 11:15(2):118-125.

471 43. Kamijo K, Miyamoto T, Ando H, Tanaka Y, Kikuchi N, Shinagawa M, Yamada S, Asaka R, Fuseya C, Ohira S, Shiozawa T. Clinical characteristics of a novel "Type 3" vasa previa: case series at a single center. *J Matern Fetal Neonatal Med. 2022*; 35:7730-7736.

For peer review only

For peer review only - http://bmjopen.bmj.com/site/about/guidelines.xhtml

475 **Figure Legends**

477 **Figure 1.** Flow chart demonstrating process of selection of studies included in the systematic review

480 **Figure 2.** Forest plot demonstrating incidence of vasa praevia in studies reporting results of routine screening in low-risk unselected pregnancies. Incidence (%; 95% confidence intervals) is reported as number of cases per 1,000 pregnancies.

For peer review only

For peer review only - http://bmjopen.bmj.com/site/about/guidelines.xhtml

Table 1. Results from meta-analysis using a random effects model to estimate summary statistics for incidence (95% confidence interval) of vasa praevia (VP) based on studies that reported VP cases along with data regarding an unselected cohort of pregnancies during the study period.

Author (year)	n/N	n/1,000 pregnancies	1 per *n* pregnancies
Lee *et al.*, 2000, [24]	18/93,874	0.19 (0.11-0.30)	5,208 (3,300-8,772)
Catanzarite *et al.*, 2001, [8]	11/33,208	0.33 (0.17-0.59)	3,201 (1,686-6,061)
Schachter *et al.*, 2002, [25]	12/72,818	0.17 (0.09-0.29)	6,061 (3,472-11,737)
Baulies *et al.*, 2007, [26]	9/12,063	0.75 (0.34-1.42)	1,340 (706-2,933)
Suzuki *et al.*, 2008, [27]	3/7,713	0.39 (0.08-1.14)	2,571 (877-12,469)
Smorgick *et al.*, 2010, [28]	19/110,684	0.17 (0.10-0.27)	5,814 (3,731-9,709)
Hasegawa *et al.*, 2010, [29]	10/4,532	2.21 (1.06-4.05)	452 (247-943)
Kanda *et al.*, 2011, [30]	10/5,131	1.95 (0.94-3.58)	513 (279-1,070)
Rebarber *et al.*, 2013, [31]	24/27,573	0.87 (0.56-1.29)	1,149 (775-1,792)
Bronsteen *et al.*, 2013, [32]	74/182,554	0.41 (0.32-0.51)	2,469 (1,965-3,145)
Hagesawa *et al.*, 2015, [33]	21/8,176	2.57 (1.59-3.92)	389 (255-629)
Catanzarite *et al.*, 2016, [10]	96/100,481	0.96 (0.77-1.17)	1,047 (857-1,292)
Kulkarni *et al.*, 2017, [34]	35/56,000	0.63 (0.44-0.87)	1,600 (1,151-2,299)
Nohuz *et al.*, 2017, [35]	8/18,152	0.44 (0.19-0.87)	2,268 (1,152-5,263)
Sullivan *et al.*, 2017, [11]	63/294,045	0.21 (0.17-0.27)	4,673 (3,650-6,061)
Yeaton-Massey *et al.*, 2019, [36]	586/945,950	0.62 (0.57-0.67)	1,616 (1,488-1,754)
Derisbourg *et al.*, 2019, [37]	4/1,620	2.47 (0.67-6.31)	405 (158-1,486)
Klahr *et al.*, 2019, [38]	61/37,236	1.64 (1.25-2.10)	610 (476-800)
La *et al.*, 2020, [39]	19/56,045	0.34 (0.20-0.53)	2,950 (1,890-4,902)
Zhang *et al.*, 2020, [12]	21/26,830	0.78 (0.49-1.20)	1,277 (836-2,062)
Gross *et al.*, 2021, [40]	21/5,905	3.56 (2.20-5.43)	281 (184-455)
Sutera *et al.*, 2021, [41]	24/89,600	0.27 (0.17-0.40)	3,731 (2,506-5,814)
Liu *et al.*, 2021, [42]	157/79,647	1.97 (1.68-2.30)	508 (435-595)
Kamijo *et al.*, 2022, [43]	14/8,723	1.60 (0.88-2.69)	625 (372-1,139)
**Pooled analysis**	**1,320/2,278,561**	**0.79 (0.59-1.01)**	**1,271 (990-1,692)**
Cochrane Q (DF)	550.11 (23)		
I^2 (inconsistency) (%) (95%CI)	95.8 (94.7-96.7)		
Egger bias (p value)	2.1799 (0.1692)		

For peer review only - http://bmjopen.bmj.com/site/about/guidelines.xhtml

488 **Table 2.** Results from meta-analysis using a random effects model to estimate summary
489 statistics for incidence (95% confidence interval) of vasa praevia (VP) based on screening
490 cohort studies that reported a specific policy of screening for VP during the study period.
491

Author (year)	n/N	n/1,000 pregnancies	1 per *n* pregnancies
Baulies *et al.*, 2007, [26]	9/12,063	0.75 (0.34-1.42)	1,340 (706-2,933)
Rebarber *et al.*, 2013, [31]	24/27,573	0.87 (0.56-1.29)	1,149 (775-1,792)
Catanzarite *et al.*, 2016, [10]	96/100,481	0.96 (0.77-1.17)	1,047 (857-1,292)
Kulkarni *et al.*, 2017, [34]	35/56,000	0.63 (0.44-0.87)	1,600 (1,151-2,299)
Nohuz *et al.*, 2017, [35]	8/18,152	0.44 (0.19-0.87)	2,268 (1,152-5,263)
Sullivan *et al.*, 2017, [11]	63/294,045	0.21 (0.17-0.27)	4,673 (3,650-6,061)
Klahr *et al.*, 2019, [38]	61/37,236	1.64 (1.25-2.10)	610 (476-800)
La *et al.*, 2020, [39]	19/56,045	0.34 (0.20-0.53)	2,950 (1,890-4,902)
Zhang *et al.*, 2020, [12]	21/26,830	0.78 (0.49-1.20)	1,277 (836-2,062)
Gross *et al.*, 2021, [40]	21/5,905	3.56 (2.20-5.43)	281 (184-455)
Sutera *et al.*, 2021, [41]	24/89,600	0.27 (0.17-0.40)	3,731 (2,506-5,814)
Kamijo *et al.*, 2022, [43]	14/8,723	1.60 (0.88-2.69)	625 (372-1,139)
**Pooled analysis**	**395/732,654**	**0.82 (0.53-1.18)**	**1,218 (847-1,901)**
Cochrane Q (DF)	222.18 (11)		
I^2 (inconsistency) (%) (95%CI)	95.1 (92.9-96.5)		
Egger bias (p value)	6.1508 (0.0092)		

492

For peer review only

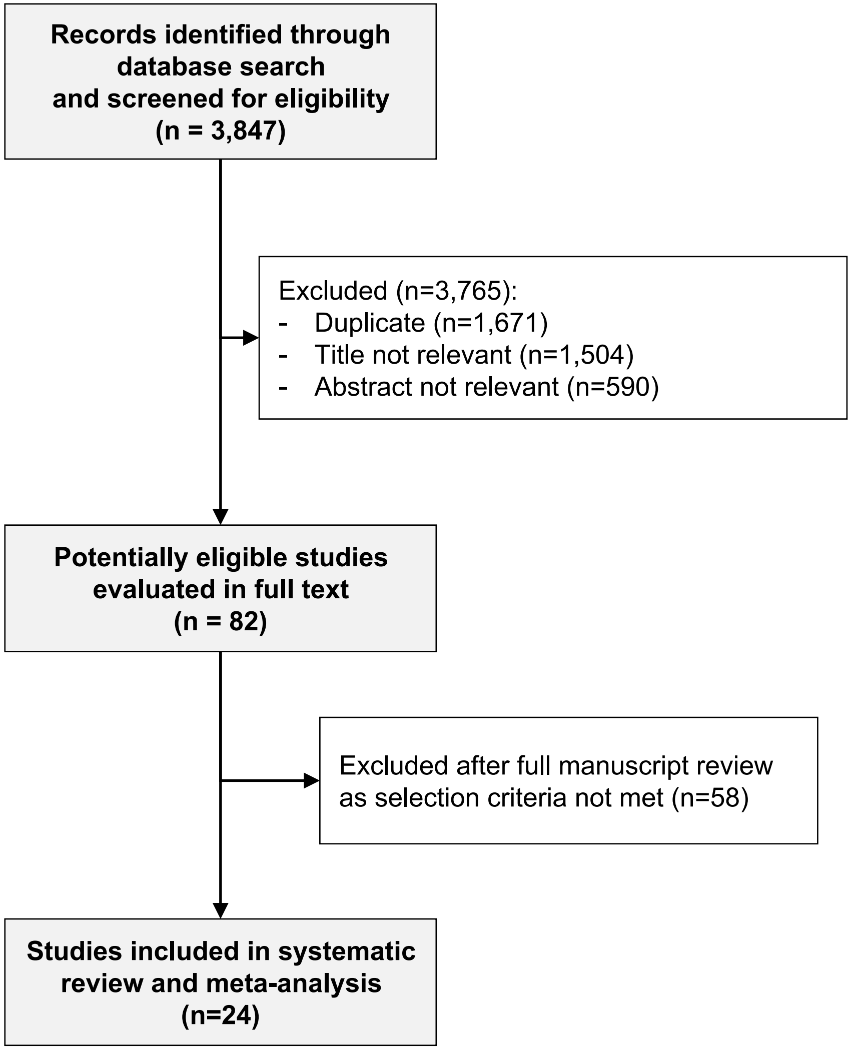
Records identified through database search and screened for eligibility (n = 3,847)
Excluded (n=3,765):
- Duplicate (n=1,671)
- Title not relevant (n=1,504)
- Abstract not relevant (n=590)
Potentially eligible studies evaluated in full text (n = 82)
Excluded after full manuscript review as selection criteria not met (n=58)
Studies included in systematic review and meta-analysis (n=24)

Figure 1.

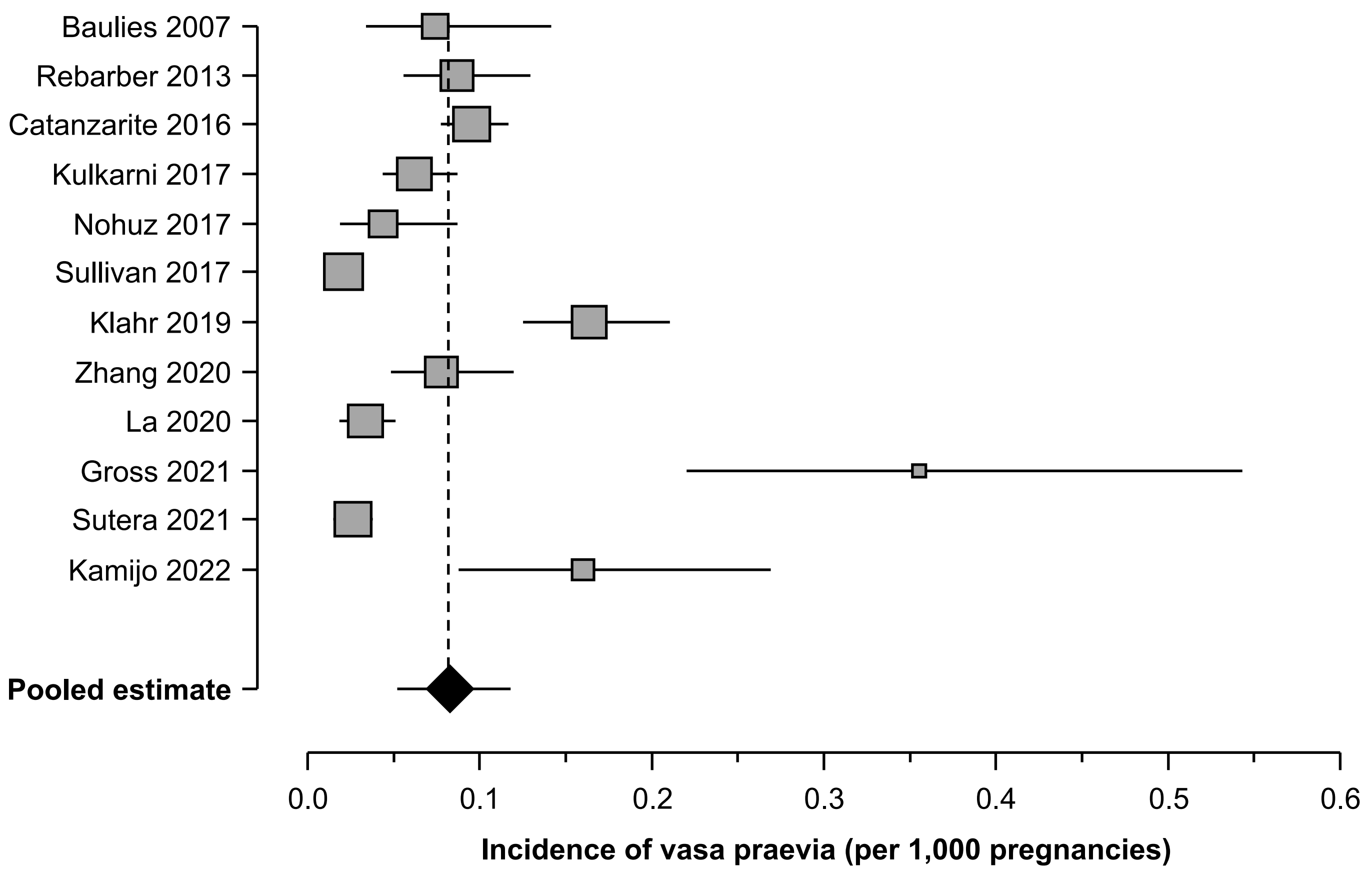

For peer review only - http://bmjopen.bmj.com/site/about/guidelines.xhtml

Figure 2.

Supplementary table S1: Search Strategy for study

**MEDLINE**
1. Medline(Vasa praevia OR vasa previa).ti,ab<br>2. Medline(abnormal cord insertion).ti,ab<br>3. Medline(marginal cord insertion).ti,ab<br>4. Medline(Velamentous cord insertion).ti,ab<br>5. Medline(bilobed placenta).ti,ab<br>6. Medline(succenturiate lobe).ti,ab<br>7. Medline(accessory lobe).ti,ab<br>8. Medline*(1 OR 2 OR 3 OR 4 OR 5 OR 6 OR 7)*
**CINAHL**
1. CINAHL(Vasa praevia OR vasa previa).ti,ab<br>2. CINAHL(abnormal cord insertion).ti,ab<br>3. CINAHL(marginal cord insertion).ti,ab<br>4. CINAHL(Velamentous cord insertion).ti,ab<br>5. CINAHL(bilobed placenta).ti,ab<br>6. CINAHL(succenturiate lobe).ti,ab<br>7. CINAHL(accessory lobe).ti,ab<br>8. CINAHL*(1 OR 2 OR 3 OR 4 OR 5 OR 6 OR 7)*
**PubMed**
1. PubMed(Vasa praevia OR vasa previa).ti,ab<br>2. PubMed(abnormal cord insertion).ti,ab<br>3. PubMed(marginal cord insertion).ti,ab<br>4. PubMed(Velamentous cord insertion).ti,ab<br>5. PubMed(bilobed placenta).ti,ab<br>6. PubMed(succenturiate lobe).ti,ab<br>7. PubMed(accessory lobe).ti,ab<br>8. PubMed*(1 OR 2 OR 3 OR 4 OR 5 OR 6 OR 7)*
**EMBASE**
1. EMBASE(Vasa praevia OR vasa previa).ti,ab

For peer review only

2. EMBASE(abnormal cord insertion).ti,ab
3. EMBASE(marginal cord insertion).ti,ab
4. EMBASE(Velamentous cord insertion).ti,ab
5. EMBASE(bilobed placenta).ti,ab
6. EMBASE(succenturiate lobe).ti,ab
7. EMBASE(accessory lobe).ti,ab
8. EMBASE*(1 OR 2 OR 3 OR 4 OR 5 OR 6 OR 7)*

For peer review only

Supplementary table 2. Methodological assessment of included studies based on Newcastle – Ottawa Score Summary

Study	Selection	Comparability	Outcome	NOS Score
Lee *et al.*, 2000[24]	★★★	★	★★★	7
Catanzarite *et al.*, 2001[8]	★★★	★	★★★	7
Schachter *et al.*, 2002[25]	★★★	★	★★★	7
Baulies *et al.*, 2007[26]	★★★	★	★★★	7
Suzuki *et al.*, 2008[27]	★★★	★	★★★	7
Smorgick *et al.*, 2010[28]	★★★	-	★★★	6
Hasegawa *et al.*, 2010[29]	★★★	★★	★★★	8
Kanda *et al.*, 2011[30]	★★★	★	★★★	7
Rebarber *et al.*, 2013[31]	★★★	★	★★★	7
Bronsteen *et al.*, 2013[32]	★★★	★	★★★	7
Hasegawa *et al.*, 2015[33]	★★★	★	★★★	7
Catanzarite *et al.*, 2016[10]	★★★	★	★★★	7
Kulkarni *et al.*, 2017[34]	★★★	★	★★★	7
Nohuz *et al.*, 2017[35]	★★★	★	★★★	7
Sullivan *et al.*, 2017[11]	★★★	★	★★★	7
Yeaton-Massey *et al.*, 2019[36]	★★	★	★★★	6
Derisbourg *et al.*, 2019[37]	★★★	★	★★★	7
Klahr *et al.*, 2019[38]	★★	★	★★★	6
La *et al.*, 2020[39]	★★★	★	★★★	7
Zhang *et al.*, 2020[12]	★★★	★	★★★	7
Gross *et al.*, 2021[40]	★★★	★	★★★	7
Sutera *et al.*, 2021[41]	★★★	★	★★★	7
Liu *et al.*, 2021[42]	★★★	★	★★★	7
Kamijo *et al.*, 2022[43]	★★★	★	★★★	7

For peer review only

Supplementary table S3. The Preferred Reporting Items for Systematic Reviews and Meta-analysis: The PRISMA checklist 2020

Section and Topic	Item #	Checklist item	Location where item is reported
**TITLE**			
Title	1	Identify the report as a systematic review.	1
**ABSTRACT**			
Abstract	2	See the PRISMA 2020 for Abstracts checklist.	2
**INTRODUCTION**			
Rationale	3	Describe the rationale for the review in the context of existing knowledge.	4
Objectives	4	Provide an explicit statement of the objective(s) or question(s) the review addresses.	4
**METHODS**			
Eligibility criteria	5	Specify the inclusion and exclusion criteria for the review and how studies were grouped for the syntheses.	5
Information sources	6	Specify all databases, registers, websites, organisations, reference lists and other sources searched or consulted to identify studies. Specify the date when each source was last searched or consulted.	5
Search strategy	7	Present the full search strategies for all databases, registers and websites, including any filters and limits used.	5 (Supplementary table 1)
Selection process	8	Specify the methods used to decide whether a study met the inclusion criteria of the review, including how many reviewers screened each record and each report retrieved, whether they worked independently, and if applicable, details of automation tools used in the process.	5
Data collection process	9	Specify the methods used to collect data from reports, including how many reviewers collected data from each report, whether they worked independently, any processes for obtaining or confirming data from study investigators, and if applicable, details of automation tools used in the process.	5
Data items	10a	List and define all outcomes for which data were sought. Specify whether all results that were compatible with each outcome domain in each study were sought (e.g. for all measures, time points, analyses), and if not, the methods used to decide which results to collect.	5
	10b	List and define all other variables for which data were sought (e.g. participant and intervention characteristics, funding sources). Describe any assumptions made about any missing or unclear information.	5,6
Study risk of bias assessment	11	Specify the methods used to assess risk of bias in the included studies, including details of the tool(s) used, how many reviewers assessed each study and whether they worked independently, and if applicable, details of automation tools used in the process.	5,6
Effect measures	12	Specify for each outcome the effect measure(s) (e.g. risk ratio, mean difference) used in the synthesis or presentation of results.	6
Synthesis	13a	Describe the processes used to decide which studies were eligible for each synthesis (e.g. tabulating the study	5

For peer review only

Section and Topic	Item #	Checklist item	Location where item is reported
methods		intervention characteristics and comparing against the planned groups for each synthesis (item #5)).	
	13b	Describe any methods required to prepare the data for presentation or synthesis, such as handling of missing summary statistics, or data conversions.	6
	13c	Describe any methods used to tabulate or visually display results of individual studies and syntheses.	6
	13d	Describe any methods used to synthesize results and provide a rationale for the choice(s). If meta-analysis was performed, describe the model(s), method(s) to identify the presence and extent of statistical heterogeneity, and software package(s) used.	6
	13e	Describe any methods used to explore possible causes of heterogeneity among study results (e.g. subgroup analysis, meta-regression).	6
	13f	Describe any sensitivity analyses conducted to assess robustness of the synthesized results.	NA
Reporting bias assessment	14	Describe any methods used to assess risk of bias due to missing results in a synthesis (arising from reporting biases).	6
Certainty assessment	15	Describe any methods used to assess certainty (or confidence) in the body of evidence for an outcome.	NA
**RESULTS**			
Study selection	16a	Describe the results of the search and selection process, from the number of records identified in the search to the number of studies included in the review, ideally using a flow diagram.	6<br>Figure 1
	16b	Cite studies that might appear to meet the inclusion criteria, but which were excluded, and explain why they were excluded.	6
Study characteristics	17	Cite each included study and present its characteristics.	6,7
Risk of bias in studies	18	Present assessments of risk of bias for each included study.	7<br>Supplementary table 2
Results of individual studies	19	For all outcomes, present, for each study: (a) summary statistics for each group (where appropriate) and (b) an effect estimate and its precision (e.g. confidence/credible interval), ideally using structured tables or plots.	7,8; Table 1, Figure 2
Results of syntheses	20a	For each synthesis, briefly summarise the characteristics and risk of bias among contributing studies.	7; Supplementary table 2
	20b	Present results of all statistical syntheses conducted. If meta-analysis was done, present for each the summary estimate and its precision (e.g. confidence/credible interval) and measures of statistical heterogeneity. If comparing groups, describe the direction of the effect.	7,8; Table 1, Figure 2
	20c	Present results of all investigations of possible causes of heterogeneity among study results.	7; Table 1

For peer review only

Section and Topic	Item #	Checklist item	Location where item is reported
	20d	Present results of all sensitivity analyses conducted to assess the robustness of the synthesized results.	NA
Reporting biases	21	Present assessments of risk of bias due to missing results (arising from reporting biases) for each synthesis assessed.	NA
Certainty of evidence	22	Present assessments of certainty (or confidence) in the body of evidence for each outcome assessed.	NA
**DISCUSSION**			
Discussion	23a	Provide a general interpretation of the results in the context of other evidence.	8,9
	23b	Discuss any limitations of the evidence included in the review.	9,10
	23c	Discuss any limitations of the review processes used.	10
	23d	Discuss implications of the results for practice, policy, and future research.	9
**OTHER INFORMATION**			
Registration and protocol	24a	Provide registration information for the review, including register name and registration number, or state that the review was not registered.	5
	24b	Indicate where the review protocol can be accessed, or state that a protocol was not prepared.	5, PROSPERO website
	24c	Describe and explain any amendments to information provided at registration or in the protocol.	NA
Support	25	Describe sources of financial or non-financial support for the review, and the role of the funders or sponsors in the review.	6,11
Competing interests	26	Declare any competing interests of review authors.	11
Availability of data, code and other materials	27	Report which of the following are publicly available and where they can be found: template data collection forms; data extracted from included studies; data used for all analyses; analytic code; any other materials used in the review.	On request from authors

For peer review only

Supplementary Table 4. Characteristics of the included studies

Author (year)	Study design	Study location	Population	Study period	VP Cases	Population screened	Characteristics and outcomes	Postnatal confirmation of VP
Baulies *et al.*, 2007[26]	Retrospective cohort study	Spain	Unselected pregnancies	2000-2005	9	12,063	Risk factors, delivery and neonatal outcomes	Yes
Rebarber *et al.*, 2013[31]	Retrospective cohort study	United States	Unselected pregnancies	2005-2012	24	27,573	Resolution rate, delivery outcomes, hospital stay	Yes
Catanzarite *et al.*, 2016[10]	Retrospective cohort study	United States	Unselected pregnancies	2003-2015	96	100,481	Risk factors, Antepartum haemorrhage, tocolysis, delivery and neonatal outcomes	Yes
Kulkarni *et al.*, 2017[34]	Retrospective cohort study	United States	Unselected pregnancies	2009-2017	35	56,000	Risk factors, Antepartum haemorrhage, delivery and neonatal outcomes	Yes
Nohuz *et al.*, 2017[35]	Retrospective cohort study	France	Unselected pregnancies	2011-2015	8	18,152	Risk factors, Antepartum haemorrhage, delivery and neonatal outcomes	Yes
Sullivan *et al.*, 2017[11]	Population cohort study	Australia	Unselected pregnancies	2013-2014	63	294,045	Risk factors, Antepartum haemorrhage, delivery and	Yes

For peer review only

							neonatal outcomes	
Klahr *et al.*, 2019[38]	Retrospective cohort study	United States	Unselected pregnancies	2005-2018	61	37,236	Maternal characteristics, placenta and vessel types	Yes
La *et al.*, 2020[39]	Retrospective cohort study	Australia	Unselected pregnancies	2007-2017	19	56,045	Maternal characteristics, Antepartum haemorrhage, Preterm ROM, delivery and neonatal outcomes	Yes
Zhang *et al.*, 2020[12]	Retrospective cohort study	United Kingdom	Unselected pregnancies	2011-2018	21	26,830	Maternal characteristics, delivery and neonatal outcomes	Yes
Gross *et al.*, 2021[40]	Retrospective cohort study	Austria	Unselected pregnancies	2018-2019	21	5,905	Maternal characteristics, delivery and neonatal outcomes	Yes
Sutera *et al.*, 2021[41]	Retrospective cohort study	Italy	Unselected pregnancies	2007-2018	24	89,600	Maternal characteristics, delivery and neonatal outcomes	Yes
Kamijo *et al.*, 2022[43]	Retrospective cohort study	Japan	Unselected pregnancies	2010-2020	14	8,723	Type of VP, gestation at delivery, Birth weight, neonatal Hb	Yes

VP = Vasa praevia; ROM = rupture of membranes

For peer review only

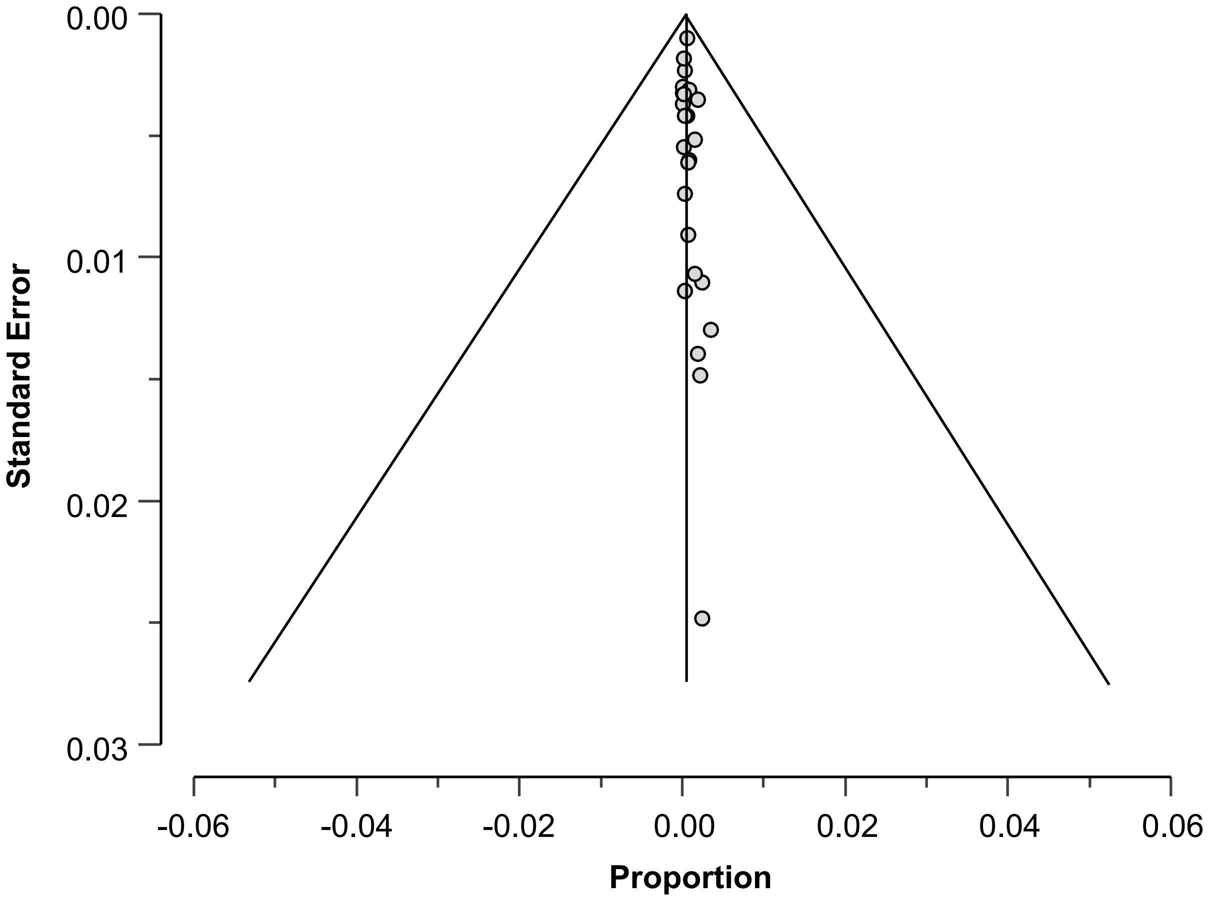

Supplementary Figure F1.

Supplementary table S3. The Preferred Reporting Items for Systematic Reviews and Meta-analysis: The PRISMA checklist 2020

Section and Topic	Item #	Checklist item	Location where item is reported
**TITLE**			
Title	1	Identify the report as a systematic review.	1
**ABSTRACT**			
Abstract	2	See the PRISMA 2020 for Abstracts checklist.	2
**INTRODUCTION**			
Rationale	3	Describe the rationale for the review in the context of existing knowledge.	4
Objectives	4	Provide an explicit statement of the objective(s) or question(s) the review addresses.	4
**METHODS**			
Eligibility criteria	5	Specify the inclusion and exclusion criteria for the review and how studies were grouped for the syntheses.	5
Information sources	6	Specify all databases, registers, websites, organisations, reference lists and other sources searched or consulted to identify studies. Specify the date when each source was last searched or consulted.	5
Search strategy	7	Present the full search strategies for all databases, registers and websites, including any filters and limits used.	5 (Supplementary table 1)
Selection process	8	Specify the methods used to decide whether a study met the inclusion criteria of the review, including how many reviewers screened each record and each report retrieved, whether they worked independently, and if applicable, details of automation tools used in the process.	5
Data collection process	9	Specify the methods used to collect data from reports, including how many reviewers collected data from each report, whether they worked independently, any processes for obtaining or confirming data from study investigators, and if applicable, details of automation tools used in the process.	5
Data items	10a	List and define all outcomes for which data were sought. Specify whether all results that were compatible with each outcome domain in each study were sought (e.g. for all measures, time points, analyses), and if not, the methods used to decide which results to collect.	5
	10b	List and define all other variables for which data were sought (e.g. participant and intervention characteristics, funding sources). Describe any assumptions made about any missing or unclear information.	5,6
Study risk of bias assessment	11	Specify the methods used to assess risk of bias in the included studies, including details of the tool(s) used, how many reviewers assessed each study and whether they worked independently, and if applicable, details of automation tools used in the process.	5,6
Effect measures	12	Specify for each outcome the effect measure(s) (e.g. risk ratio, mean difference) used in the synthesis or presentation of results.	6
Synthesis	13a	Describe the processes used to decide which studies were eligible for each synthesis (e.g. tabulating the study	5

For peer review only

Section and Topic	Item #	Checklist item	Location where item is reported
methods		intervention characteristics and comparing against the planned groups for each synthesis (item #5)).	
	13b	Describe any methods required to prepare the data for presentation or synthesis, such as handling of missing summary statistics, or data conversions.	6
	13c	Describe any methods used to tabulate or visually display results of individual studies and syntheses.	6
	13d	Describe any methods used to synthesize results and provide a rationale for the choice(s). If meta-analysis was performed, describe the model(s), method(s) to identify the presence and extent of statistical heterogeneity, and software package(s) used.	6
	13e	Describe any methods used to explore possible causes of heterogeneity among study results (e.g. subgroup analysis, meta-regression).	6
	13f	Describe any sensitivity analyses conducted to assess robustness of the synthesized results.	NA
Reporting bias assessment	14	Describe any methods used to assess risk of bias due to missing results in a synthesis (arising from reporting biases).	6
Certainty assessment	15	Describe any methods used to assess certainty (or confidence) in the body of evidence for an outcome.	NA
**RESULTS**			
Study selection	16a	Describe the results of the search and selection process, from the number of records identified in the search to the number of studies included in the review, ideally using a flow diagram.	6<br>Figure 1
	16b	Cite studies that might appear to meet the inclusion criteria, but which were excluded, and explain why they were excluded.	6
Study characteristics	17	Cite each included study and present its characteristics.	6,7
Risk of bias in studies	18	Present assessments of risk of bias for each included study.	7<br>Supplementary table 2
Results of individual studies	19	For all outcomes, present, for each study: (a) summary statistics for each group (where appropriate) and (b) an effect estimate and its precision (e.g. confidence/credible interval), ideally using structured tables or plots.	7,8; Table 1, Figure 2
Results of syntheses	20a	For each synthesis, briefly summarise the characteristics and risk of bias among contributing studies.	7; Supplementary table 2
	20b	Present results of all statistical syntheses conducted. If meta-analysis was done, present for each the summary estimate and its precision (e.g. confidence/credible interval) and measures of statistical heterogeneity. If comparing groups, describe the direction of the effect.	7,8; Table 1, Figure 2
	20c	Present results of all investigations of possible causes of heterogeneity among study results.	7; Table 1

For peer review only

Section and Topic	Item #	Checklist item	Location where item is reported
	20d	Present results of all sensitivity analyses conducted to assess the robustness of the synthesized results.	NA
Reporting biases	21	Present assessments of risk of bias due to missing results (arising from reporting biases) for each synthesis assessed.	NA
Certainty of evidence	22	Present assessments of certainty (or confidence) in the body of evidence for each outcome assessed.	NA
**DISCUSSION**			
Discussion	23a	Provide a general interpretation of the results in the context of other evidence.	8,9
	23b	Discuss any limitations of the evidence included in the review.	9,10
	23c	Discuss any limitations of the review processes used.	10
	23d	Discuss implications of the results for practice, policy, and future research.	9
**OTHER INFORMATION**			
Registration and protocol	24a	Provide registration information for the review, including register name and registration number, or state that the review was not registered.	5
	24b	Indicate where the review protocol can be accessed, or state that a protocol was not prepared.	5, PROSPERO website
	24c	Describe and explain any amendments to information provided at registration or in the protocol.	NA
Support	25	Describe sources of financial or non-financial support for the review, and the role of the funders or sponsors in the review.	6,11
Competing interests	26	Declare any competing interests of review authors.	11
Availability of data, code and other materials	27	Report which of the following are publicly available and where they can be found: template data collection forms; data extracted from included studies; data used for all analyses; analytic code; any other materials used in the review.	On request from authors

For peer review only

BMJ Open

Incidence of Vasa praevia: A systematic review and meta-analysis

Journal:	*BMJ Open*
Manuscript ID	bmjopen-2023-075245.R2
Article Type:	Original research
Date Submitted by the Author:	30-Aug-2023
Complete List of Authors:	Zhang, Weiyu; Medway NHS Foundation Trust, Medway Fetal and Maternal Medicine Centre<br>Giacchino, Tara; Medway NHS Foundation Trust, Medway Fetal and Maternal Medicine Centre<br>Chanyarungrojn, Pannapat Amy; Medway NHS Foundation Trust, Medway Fetal and Maternal Medicine Centre<br>Ionescu, Olivia; Medway NHS Foundation Trust, Medway Fetal and Maternal Medicine Centre<br>Akolekar, Ranjit; Canterbury Christ Church University, Institute of Medical Sciences; Medway NHS Foundation Trust, Medway Fetal and Maternal Medicine Centre
**Primary Subject Heading**:	Obstetrics and gynaecology
Secondary Subject Heading:	Evidence based practice
Keywords:	Prenatal diagnosis < OBSTETRICS, Fetal medicine < OBSTETRICS, Systematic Review

SCHOLARONE™
Manuscripts

I, the Submitting Author has the right to grant and does grant on behalf of all authors of the Work (as defined in the below author licence), an exclusive licence and/or a non-exclusive licence for contributions from authors who are: i) UK Crown employees; ii) where BMJ has agreed a CC-BY licence shall apply, and/or iii) in accordance with the terms applicable for US Federal Government officers or employees acting as part of their official duties; on a worldwide, perpetual, irrevocable, royalty-free basis to BMJ Publishing Group Ltd ("BMJ") its licensees and where the relevant Journal is co-owned by BMJ to the co-owners of the Journal, to publish the Work in this journal and any other BMJ products and to exploit all rights, as set out in our licence.

The Submitting Author accepts and understands that any supply made under these terms is made by BMJ to the Submitting Author unless you are acting as an employee on behalf of your employer or a postgraduate student of an affiliated institution which is paying any applicable article publishing charge ("APC") for Open Access articles. Where the Submitting Author wishes to make the Work available on an Open Access basis (and intends to pay the relevant APC), the terms of reuse of such Open Access shall be governed by a Creative Commons licence – details of these licences and which Creative Commons licence will apply to this Work are set out in our licence referred to above.

Other than as permitted in any relevant BMJ Author's Self Archiving Policies, I confirm this Work has not been accepted for publication elsewhere, is not being considered for publication elsewhere and does not duplicate material already published. I confirm all authors consent to publication of this Work and authorise the granting of this licence.

For peer review only

Title: Incidence of Vasa praevia: A systematic review and meta-analysis

Authors: Weiyu Zhang[1], Tara Giacchino[1], Pannapat Amy Chanyarungrojn[1], Olivia Ionescu[1], Ranjit Akolekar[1,2]

Key words: Vasa praevia, Incidence, Stillbirths, Prenatal diagnosis

Short Title: Incidence of vasa praevia

Affiliation:

1. Medway Fetal and Maternal Medicine Centre, Medway Maritime Hospital, United Kingdom
2. Institute of Medical Sciences, Canterbury Christ Church University, Rowan William's Court, Kent, United Kingdom

Corresponding author:

Professor R Akolekar, MD, MRCOG
Institute of Medical Sciences,
Canterbury Christ Church University,
Rowan William's Court,
Chatham, Kent ME4 4UF
Email: ranjit.akolekar@nhs.net

Word count: 3,144

For peer review only

ABSTRACT

Objectives: To derive accurate estimates of the incidence of vasa praevia (VP) in a routine population of unselected pregnancies.

Design: Systematic review and meta-analysis

Data sources: A search of MEDLINE, EMBASE, CINHAL and the Cochrane database was performed to review relevant citations reporting outcomes in pregnancies with VP from January 2000 till 5th April 2023.

Eligibility criteria for selection of studies: Prospective or retrospective cohort or population studies that provided data regarding VP cases in routine unselected pregnancies during the study period. We included studies published in the English language after the year 2000 to reflect contemporary obstetric and neonatal practice.

Data extraction and synthesis: Two reviewers independently screened the retrieved citations and extracted data. The methodological quality of studies was assessed using the Newcastle-Ottawa Scale (NOS) and PRISMA (Preferred Reporting Items for Systematic reviews and Meta-Analyses) was used to ensure standardised reporting of studies.

Results: A total of 3,847 citations were screened and 82 full-text manuscripts were retrieved for analysis. There were 24 studies that met inclusion criteria, of which 12 studies reported prenatal diagnosis with a systematic protocol of screening. There were 1,320 pregnancies with VP in a total population of 2,278,561 pregnancies; the weighted pooled incidence of VP was 0.79 (95%CI: 0.59-1.01) per 1,000 pregnancies, corresponding to 1 case of VP per 1,271 (95% CI: 990-1,692) pregnancies. Nested sub-analysis of studies reporting screening for VP based on a specific protocol identified 395 pregnancies with VP in a population of 732,654 pregnancies with weighted pooled incidence of 0.82 (95%CI: 0.53-1.18) per 1,000 pregnancies (1 case of VP per 1,218 (95% CI: 847-1,901) pregnancies.

Conclusion: The incidence of VP in unselected pregnancies is 1 in 1,218 pregnancies. This is higher than is previously reported and can be used as a basis to assess whether screening for this condition should be part of routine clinical practice. Incorporation of strategies to screen for VP in routine clinical practice is likely to prevent 5% of stillbirths.

PROSPERO registration number: CRD42020125495.

For peer review only

For peer review only - http://bmjopen.bmj.com/site/about/guidelines.xhtml

STRENGTHS AND LIMITATIONS OF THE STUDY

- Our study provides an accurate estimate of incidence of vasa praevia based on a systematic review of studies with an *a priori* designed protocol registered on international register of systematic reviews (PROSPERO).
- The study only includes cohort studies providing data regarding vasa praevia and control pregnancies delivered during the study period.
- We only included studies that were published since the year 2000 to reflect contemporary clinical practice.
- We undertook a sub-group nested analysis in studies that reported a systematic and specific policy of screening for vasa praevia in their hospital and reported both cases of vasa praevia as well as the population of control pregnancies.
- A limitation of our study is that we did not provide separate incidence for singleton and multiple pregnancies.

For peer review only

INTRODUCTION

Vasa praevia (VP) is defined as presence of fetal blood vessels, arterial or venous, unsupported by placental tissue or umbilical cord traversing the amniotic membranes in the lower uterine segment in close proximity to the internal cervical os, [1-5]. These unsupported fetal vessels can be damaged either during the antenatal period or in labour leading to severe hypovolemic shock and haemorrhagic fetal death, [4,6,7]. There is considerable evidence suggesting that prenatal diagnosis of VP improves perinatal survival and lack of antenatal detection is associated with a high-risk of stillbirths, neonatal deaths and an increased risk of hypoxic morbidity in the survivors, [8-13]. Despite the compelling evidence in favour of prenatal diagnosis in improving pregnancy outcomes in pregnancies with VP, there are currently no recommendations for screening for VP in routine clinical practice, [14]. The United Kingdom (UK) National Screening Committee examined the evidence with regard to screening for VP and reported that there was low volume of evidence on the epidemiology of VP or its outcomes in the UK to justify screening, [15].

A systematic review examined the incidence of VP and reported that there was a huge variation in studies reporting VP incidence ranging from 0.19-2.21 per 1,000 pregnancies (1 per 452 to 1 per 5,882 pregnancies) with a mean estimated incidence of 0.6 per 1,000 pregnancies (1 per 1,666 pregnancies), [16]. This systematic review had significant heterogeneity and variation as it included not just cohort studies but case-control studies and those from high-risk populations. The estimated incidence in this systematic review is likely to have been underestimated as many of the included studies reported data from 80s and 90s when VP was more likely to be an incidental rather than an intentional diagnosis. In a recent large prospective observational screening study of more than 26,000 pregnancies, we demonstrated the impact of routine screening for VP based on a 2-stage screening strategy and reported that the incidence of VP was about 1 in 1,277 pregnancies, [12]. The protocol states that stage 1 includes checking for umbilical cord insertion and placental location; those that are velamentous cord insertions in lower segment and those with a low-lying placenta are classed as at-risk and stage 2 would include a transvaginal assessment with colour Doppler ultrasound to check for presence of any fetal vessels in the vicinity of the cervix for a diagnosis of VP, [12].

The objective of this study was to undertake a systematic review of literature and undertake a meta-analysis to estimate an accurate incidence of VP in a routine population of unselected pregnancies based on analysis of studies that reported a policy of routinely screening for VP.

For peer review only - http://bmjopen.bmj.com/site/about/guidelines.xhtml

METHODS

Data sources and search strategy

This systematic review and meta-analysis was undertaken based on an *a-priori* designed study protocol recommended for systematic reviews and meta-analyses, [17]. The study protocol for the systematic review was registered in advance with the PROSPERO International prospective register of systematic reviews (Registration number: CRD42020125495) as a study to investigate perinatal outcomes as well as incidence of VP. An electronic search of MEDLINE, EMBASE and The Cochrane Library was carried out on 30th October 2022 and repeated on 5th April 2023 utilising combinations of the relevant Medical Subject Heading (MeSH) terms, key words and word variants for "vasa praevia", "abnormal cord insertion", "velamentous cord", marginal cord", "bilobed placenta" and "succenturiate lobe". The search and selection criteria were restricted to studies reported in the English language and are outlined in Supplementary table S1.

Selection criteria for studies and data extraction

The studies eligible for inclusion in this study were prospective or retrospective cohort studies and population-based studies reporting cases with VP in which the authors reported data about the total screened population during the study period. We only included studies published after 2000 to reflect contemporary clinical practice. We did a nested sub-analysis for studies which reported in their methodology that they had a routine screening policy for assessing umbilical cord insertion and VP. The citations were examined by two reviewers (WZ and PC) to produce a list of relevant studies after exclusion of duplicates, those that did not fit selection criteria after review of title and abstract, those that were case-reports, letter to editors, review articles or conference abstracts and those that were case series or case-control studies. The reference lists of relevant articles were searched, and any inconsistencies were resolved by discussion with an internal third reviewer (RA).

The citations retrieved following this search strategy were examined for relevance to this study to investigate incidence based on above eligibility criteria and in each study, we extracted information about authors, study location, years of enrolment for cases and controls, study design, sample size, whether the study was single or multi-centre, whether the study was in high-risk population or unselected general pregnant population, and whether there was a routine policy of screening for umbilical cord insertion and VP during the study period in the reported studies. Data extracted for each study was inputted into contingency tables.

For peer review only - http://bmjopen.bmj.com/site/about/guidelines.xhtml

Quality Assessment

The methodological quality of studies included in the review was assessed using the Newcastle-Ottawa Scale (NOS), [18]. We used PRISMA (Preferred Reporting Items for Systematic reviews and Meta-Analyses) to ensure standardised reporting of studies in our systematic review. The PRISMA statement for this study included a checklist and a flow chart to allow uniform and transparent reporting of the systematic review and meta-analysis, [19].

Estimation of summary statistics for incidence of vasa praevia (VP)

Data were entered in contingency tables for cases and control pregnancies to calculate weighted pooled incidence (95% confidence intervals [CI]). The incidence of VP was reported as total number of cases per 1,000 pregnancies as well as total number of pregnancies for every 1 case of VP. Summary statistics (95%CI) from each study were then combined to obtain a pooled estimate which was calculated as a weighted average of the individual study estimates. The pooled summary statistics were estimated using random-effects model (REM) to allow for assessment of between-study variability in results by weighting studies using a combination of their own variance as well as the between-study variance, [20]. The heterogeneity between studies was estimated using Cochrane's heterogeneity statistic Q, and the I^2 statistic. Regression analysis was undertaken to examine factors providing a significant contribution to the prediction of incidence of VP. We also estimated the potential impact of introducing a policy for screening for VP in preventing stillbirths in the UK based on annual number of total births and stillbirths reported by the Office of National Statistics, National Records of Scotland and Northern Ireland Statistics and Research Agency, [21-23]. The statistical software package StatsDirect version 2.7.9 (StatsDirect Ltd, Cheshire, United Kingdom) and MedCalc Statistical Software version 16.4.3 (MedCalc Software, Ostend, Belgium) were used for data analysis.

Patient and public involvement

No patients were involved in setting the research question, in developing plans for design, interpretation, reporting or implementation of the study.

RESULTS

Data search results

The electronic search from the databases yielded 3,847 potential citations; of these, we excluded 1,671 citations as they were duplicates, 1,504 after review of study title and 590 citations after review of the abstract. We retrieved 82 manuscripts in full text for detailed

For peer review only

assessment and excluded a further 58 studies that did not meet the selection criteria, thus finally including 24 studies for systematic review and meta-analysis. The search criteria are outlined in supplementary table S1, and the study selection process is outlined in the flow chart in Figure 1.

Characteristics of studies included in the systematic review

There were 24 studies included in the systematic review, [8,10-12,24-43], which provided data regarding incidence of VP. There were 7 cohort studies published in the first decade (2000 to 2010), [8,24-29] and 17 studies in the second decade (2011 to 2020), [10-12,30-43]. The published studies were from across different geographical areas; 8 studies from United states, [8,10,24,31,32,34,36,38] 5 studies from Japan, [27,29,30,33,43], 6 from Europe, [12,26,35,37,40,41], 2 each from Australia, [11,39] and Israel, [25,28] and 1 from China, [42]. All 24 studies reported the total screened population during the study period and the number of cases of VP detected, thus providing a basis of estimating incidence of VP. Screening studies which report a policy-based assessment of an unselected cohort of pregnancies are more likely to reflect the true incidence of VP, compared to studies that included an incidental diagnosis of VP. Therefore, we identified studies that described a specific protocol for assessment of all pregnancies during the study period for prenatal diagnosis of VP after excluding those that had a variable protocol for identification of at-risk pregnancies, those that were carried out in a specific high-risk population such as those with velamentous cord insertion, [37], succenturiate lobe, [27], *in vitro* fertilisation pregnancies, [25], or those who were only delivered by caesarean section, [36]. After exclusion of above studies, we identified 12 studies, [10-12,26,31,34,35,38-41,43], in which the authors reported that they had a systematic protocol to document the placental location and umbilical cord insertion at the first or second trimester scan; those that were suspected to have VP were further scanned with transvaginal ultrasound to confirm the diagnosis. In the 12 screening protocol-based studies included, the authors reported confirmation of the presence of VP postnatally (Supplementary table S2).

Assessment of quality and heterogeneity of studies

The methodological quality of studies included in this systematic review was assessed using the NOS. The rating of the included studies according to the NOS based on selection, comparability and outcome are shown in Supplementary table S3. The PRISMA guidance was followed for reporting this meta-analysis and is reported in Supplementary table S4). The funnel plot of included studies demonstrating the publication bias is shown in Supplementary figure F1.

For peer review only - http://bmjopen.bmj.com/site/about/guidelines.xhtml

Incidence of vasa praevia (VP)

There were 24 studies that reported data about cases with VP with a corresponding cohort of total number of unselected pregnancies screened in a routine population during the study period, [8,10-12,24-43]. There were 1,320 pregnancies with VP in a total population of 2,278,561 pregnancies. The weighted pooled incidence of VP was 0.79 (95%CI: 0.59-1.01) per 1,000 pregnancies screened, which corresponded to an incidence of 1 case of VP per 1,271 (95% CI: 990-1,692) pregnancies (Table 1).

There were 12 studies that reported data regarding incidence of VP along with the total pregnancies screened based on a specific screening protocol, [10-12,26,31,34,35,38-41,43]. There were 395 pregnancies with VP in a total population of 732,654 pregnancies. The weighted pooled incidence of VP was 0.82 (95%CI: 0.53-1.18) per 1,000 pregnancies screened, which corresponded to an incidence of 1 case of VP per 1,218 (95% CI: 847-1,901) pregnancies (Table 2, Figure 2).

Regression analysis demonstrated that in prediction of the incidence of VP, there was a significant independent contribution from the publication date of the study, whether the study was published in the first decade (2000-2010) or in the second decade (2011-2022) ($p<0.011$) but there was no significant association with the country in which the study was conducted ($p=0.408$) ($R^2=0.208$; $p<0.05$). There was a significant increase in incidence of VP from studies published in the second decade (2011-2022) compared to first decade (2000-2010) (OR: 2.60; 95%CI: 2.08-3.25) ($p<0.01$).

Potential impact on prevention of stillbirths in United Kingdom

There were total of 697,551 births in the UK with 2,866 (0.41%) stillbirths in 2021. Given an incidence of VP of 1 in 1,218 pregnancies, there would be an estimated 573 pregnancies with VP per year in the UK. Based on estimates from the systematic review and meta-analysis reporting perinatal survival in pregnancies with VP, the livebirth rate in pregnancies with VP with and without prenatal diagnosis is 98.6% (95%CI: 96.7-99.7) and 72.1% (95%CI: 50.6-89.4), respectively; therefore, the expected number of stillbirths in pregnancies with VP in those with and without prenatal diagnosis would be 8 (95%CI: 2-19) and 160 (95% CI: 61-283). Therefore, the potential impact of introducing a policy of screening for VP in clinical practice could contribute to prevention of 5.30% (95%CI: 2.11-9.89) of stillbirths in the UK. This is based on the assumption that VP is causal to the fetal death but in some pregnancies with VP, other risk factors and / or pregnancy complications may also play a role.

For peer review only - http://bmjopen.bmj.com/site/about/guidelines.xhtml

DISCUSSION

Principal findings of the study

The findings of our systematic review and meta-analysis demonstrate that the incidence of VP in an unselected population of pregnancies is 1 in 1,218 pregnancies. The estimated incidence of VP in our study is based on weighted pooled estimates calculated firstly, from meta-analysis of studies published in the last 20 years to reflect contemporary obstetric and neonatal practice and secondly, by inclusion of those studies that reported a specific policy of assessment of placental location and umbilical cord insertion for prenatal diagnosis of VP in their routine practice. The incidence of VP was not affected by the study location or where the study was carried out, reflecting that in centres that have a specific protocol for assessment of VP as described above, there is no significant variation in reported incidence. Our study demonstrates that if screening for prenatal diagnosis of VP was introduced in routine clinical practice, it can potentially prevent 5% of stillbirths.

Comparison with previous studies

The results of our study demonstrate that VP is not an uncommon obstetric complication and has an incidence of 1 in 1,218 pregnancies, which is higher than that reported in previous studies, [16]. A previous systematic review reported a wide variation in the incidence of VP from 1 per 452 to 5,882 pregnancies with a mean incidence of 1 in 1,666 pregnancies. The authors included a wide range of studies including case-control studies, retrospective cohort studies, studies carried out on high-risk populations (such as those with pregnancies conceived with *in vitro* fertilisation, studies examining outcomes in pregnancies with bilobed placenta or in those that had a velamentous cord insertion), those that reported an incidental diagnosis of VP rather than cases detected in an unselected population based on a systematic protocol of screening and inclusion of studies that did not report whether there was postnatal confirmation of prenatal findings of VP, thus leading to a wide heterogeneity in the results and potentially underestimation of the incidence. Moreover, the authors included studies reporting data from 80s and 90s, which are more likely to be incidentally diagnosed cases of VP rather than as a result of intentional screening.

Implications for clinical practice

Our study highlights that the incidence of VP is much higher than is previously reported. The results of our study provide an accurate estimate of the incidence which can for the basis for further studies to investigate whether VP screening should be incorporated in routine practice as not only it is more common than previously believed, but more importantly, prenatal diagnosis can prevent adverse outcomes associated with VP. There is robust and unequivocal

For peer review only - http://bmjopen.bmj.com/site/about/guidelines.xhtml

evidence from several studies, including our cohort study, suggesting that prenatal diagnosis of VP is associated with high rates of livebirths and perinatal survival, [10-12,30,34,35,40-42]. A recent systematic review and meta-analysis by our group summarised and confirmed the results from previous studies demonstrating that prenatal diagnosis of VP is associated with a 99% rate of perinatal survival but a lack of an antenatal diagnosis increases the risk of a perinatal hypoxic morbidity 50-fold and that of perinatal death by 25-fold, [13]. There are few other examples in obstetric practice which demonstrate such a significant and stark impact of prenatal diagnosis on improving pregnancy outcomes such as the prenatal diagnosis of VP. There are 12 relatively recent studies, [10-12,26,31,34,35,38-41,43], which report that systematic and routine screening for VP is feasible in routine clinical practice; in fact, our recent prospective observational study of more than 26,000 pregnancies demonstrates that a 2-stage screening strategy for VP can be easily implemented in routine clinical practice and such a policy is associated with a high rate of prenatal diagnosis and good perinatal outcomes, [12]. The recommendations for screening for VP in routine practice is hindered by evidence about accurate incidence in routine pregnant population. Our study should pave the way for further studies that investigate strategies of introduction screening for VP in routine pregnancies.

Strengths and limitations

The strength of our study is that it summarises results of all studies reported in the last two decades and provides accurate summary estimates for incidence of VP in current obstetric practice. We used a standardised methodology for conducting this systematic review and meta-analysis with design of an *a-priori* protocol, registration with the international register at PROSPERO, a comprehensive search strategy, appropriate quality assessment of studies included in systematic review with NOS and standardised reporting of systematic review with PRISMA checklist. The limitations of our study relate to standard biases associated with meta-analysis such as inclusion of studies with different sample size, methodology and design which may introduce heterogeneity in the analysis. However, as a means to minimize the effects of such variation and heterogeneity, we reviewed all these studies in detail to select and identify those studies that provide data from low-risk unselected pregnancies rather than high-risk populations. This allowed us to do a nested meta-analysis to estimate an accurate weighted pooled summary statistic, which is more reflective of true population incidence rather than just a pooled estimated from all reported studies. We used a random effects model to mitigate the impact of heterogeneity as this model allows for assessment of between-study variability in results by weighing studies using a combination of their own variance as well as the between-study variance. Furthermore, we performed regression analyses to examine temporal and spatial trends in VP incidence such as the publication dates as well as the location of where the studies were carried out. A confounding factor and a limitation of our study is that we did

For peer review only - http://bmjopen.bmj.com/site/about/guidelines.xhtml

not exclude multiple pregnancies and this incidence is the total incidence in singleton and multiple pregnancies; the incidence in multiple pregnancies could potentially be higher but requires further studies to investigate this.

Conclusions

VP is not an uncommon obstetric complication. The incidence of VP in an unselected population is 1 in 1,218 pregnancies, which is considerably higher than previously reported estimates. Further research studies should be undertaken to model and investigate strategies for incorporating prenatal screening for VP in routine clinical practice, given the feasibility of screening and major impact on prevention of stillbirths and hypoxic morbidity with prenatal diagnosis of VP.

Acknowledgement: We are grateful to the Medway Library and Knowledge Service for their help and assistance with search strategy. This study is a part of the PhD thesis of Weiyu Zhang for the Canterbury Christ Church University.

Author statement: WZ, PC, TG and OI contributed to literature search, study selection, data extraction and assessment of study quality. WZ and RA wrote the initial and final draft of the manuscript. RA conceived the study and supervised the data extraction, discussed studies for inclusion following initial review and agreed the final draft of the manuscript for submission. All authors reviewed the initial and final draft of the manuscript and contributed to the intellectual content.

Data availability statement: Data are available upon reasonable request.

Ethics approval: No ethical approval was required for this study.

Conflict of interest: None

Funding: No funding was received for this study.

For peer review only - http://bmjopen.bmj.com/site/about/guidelines.xhtml

REFERENCES

1. McNair AJ. Placenta praevia, with vasa praevia; Caesarean section. *Proc R Soc Med* 1921; 14: 195-196.
2. Alment EA. Vasa praevia simulating antepartum hemorrhage. *Br Med J* 1949; 2: 1273.
3. Sinkey RG, Odibo AO, Dashe JS. Diagnosis and management of vasa previa. Society of Maternal-Fetal (SMFM) Publications Committee. *Am J Obstet Gynecol* 2015; 213: 615-619.
4. Oyelese KO, Turner M, Lees C, Campbell S. Vasa previa: an avoidable obstetric tragedy. *Obstet Gynecol Surv* 1999; 54: 138–45.
5. Catanzarite V, Oyelese Y. Diagnosis and management of vasa previa. *Am J Obstet Gynecol* 2016; 214: 764.
6. Robert JA, Sepulveda W. Fetal exsanguination from ruptured vasa previa: still a catastrophic event in modern obstetrics. *J Obstet Gynaecol* 2003; 23: 574.
7. Antoine C, Young BK, Silverman F, Greco MA,lvarez SP. Sinusoidal fetal heart rate pattern with vasa previa in twin pregnancy. *J Reprod Med* 1982; 27: 295–300.
8. Catanzarite V, Maida C, Thomas W, Mendoza A, Stanco L, Piacquadio KM. Prenatal sonographic diagnosis of vasa previa: ultrasound findings and obstetric outcome in ten cases. *Ultrasound Obstet Gynecol* 2001; 18:109-115.
9. Oyelese Y, Catanzarite V, Prefumo F, Lashley S, Schachter M, Tovbin Y, Goldstein V, Smulian JC. Vasa previa: the impact of prenatal diagnosis on outcomes. *Obstet Gynecol* 2004; 103: 937-942.
10. Catanzarite V, Cousins L, Daneshmand S, Schwendemann W, Casele H, Adamczak J, Tith T, Patel A. Prenatally Diagnosed Vasa Previa: A Single-Institution Series of 96 Cases. *Obstet Gynecol* 2016; 128: 1153-1161.
11. Sullivan EA, Javid N, Duncombe G, Li Z, Safi N, Cincotta R, Homer CSE, Halliday L, Oyelese Y. Vasa Previa Diagnosis, Clinical Practice, and Outcomes in Australia. *Obstet Gynecol* 2017; 130: 591-598.
12. Zhang W, Geris S, Beta J, Ramadan G, Nicolaides KH, Akolekar R. Prevention of stillbirth: impact of two-stage screening for vasa previa. *Ultrasound Obstet Gynecol*. 2020; 55: 605-612.
13. Zhang W, Geris S, Al-Emara N, Ramadan G, Sotiriadis A, Akolekar R. Perinatal survival in pregnancies with a prenatal diagnosis of vasa praevia: a systematic review and meta-analysis. *Ultrasound Obstet Gynecol*. 2021; 57: 710-719.
14. Jauniaux E, Alfirevic Z, Bhide AG, Burton GJ, Collins SL, Silver R; Royal College of Obstetricians and Gynaecologists. Vasa Praevia: Diagnosis and Management: Green-top Guideline No. 27b. *BJOG*. 2019; 126: e49-e61.

For peer review only - http://bmjopen.bmj.com/site/about/guidelines.xhtml

15. Screening for Vasa praevia: An evidence map to outline the volume and type of evidence related to screening for vasa praevia for the UK National Screening Committee: Version 1.1: Costello Medical: UK National Screening Committee. March 2023. Vasa praevia - UK National Screening Committee (UK NSC) - GOV.UK (view-health-screening-recommendations.service.gov.uk). Accessed 28th April 2023.

16. Ruiter L, Kok N, Limpens J, Derks JB, de Graaf IM, Mol B, Pajkrt E. Incidence of and risk indicators for vasa praevia: a systematic review. *BJOG.* 2016; 23: 1278-87.

17. NHS Centre for Reviews and Dissemination. Systematic reviews: CRD's guidance for undertaking reviews in health care. University of York: York, UK, 2009.

18. Wells GA, Shea B, O'Connell D, Peterson J, Welch V, Losos M, Tugwell P. The Newcastle-Ottawa Scale (NOS) for assessing the quality of nonrandomized studies in meta-analysis. http://www.ohri.ca/programs/clinical_epidemiology/oxford.asp.

19. Moher D, Liberati A, Tetzlaff J, Altman DG, The PRISMA Group. Preferred Reporting Items for Systematic Reviews and Meta-Analyses: The PRISMA Statement. *BMJ* 2009; 39: b2535.

20. DerSimonian R, Laird N: Meta-analysis in clinical trials. *Control Clin Trials* 1986; 7: 177-188.

21. Births in England and Wales: Summary tables: August 2022. https://www.ons.gov.uk/peoplepopulationandcommunity/birthsdeathsandmarriages/livebirths/bulletins/birthsummarytablesenglandandwales/2021. Accessed 28th April 2023.

22. Vital events reference tables. National records of Scotland. June 2022. https://www.nrscotland.gov.uk/statistics-and-data/statistics/statistics-by-theme/vital-events/general-publications/vital-events-reference-tables/2021. Accessed 28th April 2023.

23. Stillbirths: Northern Ireland Statistics and Research Agency. November 2022. https://www.nisra.gov.uk/publications/stillbirth-and-infant-death-numbers. Accessed 28th April 2023.

24. Lee W, Lee VL, Kirk JS, Sloan CT, Smith RS, Comstock CH. Vasa previa: prenatal diagnosis, natural evolution, and clinical outcome. *Obstet Gynecol* 2000; 95: 572-576.

25. Schachter M, Tovbin Y, Arieli S, Friedler S, Ron-El R, Sherman D. In vitro fertilisation is a risk factor for vasa praevia. *Fertil Steril*. 2002; 78:642-643.

26. Baulies S, Maiz N, Muñoz A, Torrents M, Echevarría M, Serra B. Prenatal ultrasound diagnosis of vasa praevia and analysis of risk factors. *Prenat Diagn* 2007; 27: 595-599.

27. Suzuki S, Igarashi M. Clinical significance of pregnancies with succenturiate lobes of placenta. *Arch Gynecol Obstet*. 2008; 277:299-301.

For peer review only - http://bmjopen.bmj.com/site/about/guidelines.xhtml

28. Smorgick N, Tovbin Y, Ushakov F, Vaknin Z, Barzilay B, Herman A, Maymon R Is neonatal risk from vasa previa preventable? The 20-year experience from a single medical center. *J Clin Ultrasound* 2010; 38: 118-22.

29. Hasegawa J, Farina A, Nakamura M, Matsuoka R, Ichizuka K, Sekizawa A, Okai T. Analysis of the ultrasonographic findings predictive of vasa previa. *Prenat Diagn*. 2010; 30: 1121-1125.

30. Kanda E, Matsuda Y, Kamitomo M, Maeda T, Mihara K, Hatae M. Prenatal diagnosis and management of vasa previa: a 6-year review. *J Obstet Gynaecol Res* 2011; 37: 1391-1396.

31. Rebarber A, Dolin C, Fox NS, Klauser CK, Saltzman DH, Roman AS. Natural history of vasa previa across gestation using a screening protocol. *J Ultrasound Med* 2014; 33: 141-147.

32. Bronsteen R, Whitten A, Balasubramanian M, Lee W, Lorenz R, Redman M, Goncalves L, Seubert D, Bauer S, Comstock C. Vasa previa: clinical presentations, outcomes, and implications for management. *Obstet Gynecol*. 2013; 122: 352-357.

33. Hasegawa J, Arakaki T, Ichizuka K, Sekizawa A. Management of vasa previa during pregnancy. *J Perinat Med*. 2015; 43: 783-784.

34. Kulkarni A, Powel J, Aziz M, Shah L, Lashley S, Benito C, Oyelese Y. Vasa Previa: prenatal diagnosis and outcomes: thirty-five cases from a single maternal-fetal medicine practice. *J Ultrasound Med* 2018; 37: 1017-1024.

35. Nohuz E, Boulay E, Gallot D, Lemery D, Vendittelli F. Can we perform a prenatal diagnosis of vasa previa to improve its obstetrical and neonatal outcomes? *J Gynecol Obstet Hum Reprod*. 2017; 46: 373-377.

36. Yeaton-Massey A, Girsen AI, Mayo JA, Blumenfeld YJ, El-Sayed YY, Stevenson DK, Shaw GM. Vasa previa and extreme prematurity: a population-based study. *J Perinatol. 2019;* 39:475-480.

37. Derisbourg S, Boulay A, Lamy C, Barlow P, Van Rysselberge M, Thomas D, Rozenberg S, Daelemans C. First trimester ultrasound prediction of velamentous cord insertions: a prospective study. *J Matern Fetal Neonatal Med. 2021*; 34:2642-2648.

38. Klahr R, Fox NS, Zafman K, Hill MB, Connolly CT, Rebarber A. Frequency of spontaneous resolution of vasa previa with advancing gestational age. *Am J Obstet Gynecol. 2019*; 221:646.e1-646.e7.

39. La S, Melov SJ, Nayyar R. Are we over-diagnosing vasa praevia? The experience and lessons learned in a tertiary centre. *Aust N Z J Obstet Gynaecol. 2021*; 61:217-222.

40. Gross A, Markota Ajd B, Specht C, Scheier M. Systematic screening for vasa previa at the 20-week anomaly scan. *Acta Obstet Gynecol Scand. 2021;* 100:1694-1699.

For peer review only

For peer review only - http://bmjopen.bmj.com/site/about/guidelines.xhtml

41. Sutera M, Garofalo A, Pilloni E, Parisi S, Alemanno MG, Menato G, Sciarrone A, Viora E. Vasa previa: when antenatal diagnosis can change fetal prognosis. *J Perinat Med. 2021*; 49: 915-922.

42. Liu N, Hu Q, Liao H, Wang X, Yu H. Vasa previa: Perinatal outcomes in singleton and multiple pregnancies*. Biosci Trends. 2021*; 11:15(2):118-125.

43. Kamijo K, Miyamoto T, Ando H, Tanaka Y, Kikuchi N, Shinagawa M, Yamada S, Asaka R, Fuseya C, Ohira S, Shiozawa T. Clinical characteristics of a novel "Type 3" vasa previa: case series at a single center. *J Matern Fetal Neonatal Med. 2022*; 35:7730-7736.

For peer review only

Figure Legends

Figure 1. Flow chart demonstrating process of selection of studies included in the systematic review

Figure 2. Forest plot demonstrating incidence of vasa praevia in studies reporting results of routine screening in low-risk unselected pregnancies. Incidence (%; 95% confidence intervals) is reported as number of cases per 1,000 pregnancies.

For peer review only

For peer review only - http://bmjopen.bmj.com/site/about/guidelines.xhtml

Table 1. Results from meta-analysis using a random effects model to estimate summary statistics for incidence (95% confidence interval) of vasa praevia (VP) based on studies that reported VP cases along with data regarding an unselected cohort of pregnancies during the study period.

**Author (year)**	**n/N**	**n/1,000 pregnancies**	**1 per *n* pregnancies**
Lee *et al.*, 2000, [24]	18/93,874	0.19 (0.11-0.30)	5,208 (3,300-8,772)
Catanzarite *et al.*, 2001, [8]	11/33,208	0.33 (0.17-0.59)	3,201 (1,686-6,061)
Schachter *et al.*, 2002, [25]	12/72,818	0.17 (0.09-0.29)	6,061 (3,472-11,737)
Baulies *et al.*, 2007, [26]	9/12,063	0.75 (0.34-1.42)	1,340 (706-2,933)
Suzuki *et al.*, 2008, [27]	3/7,713	0.39 (0.08-1.14)	2,571 (877-12,469)
Smorgick *et al.*, 2010, [28]	19/110,684	0.17 (0.10-0.27)	5,814 (3,731-9,709)
Hasegawa *et al.*, 2010, [29]	10/4,532	2.21 (1.06-4.05)	452 (247-943)
Kanda *et al.*, 2011, [30]	10/5,131	1.95 (0.94-3.58)	513 (279-1,070)
Rebarber *et al.*, 2013, [31]	24/27,573	0.87 (0.56-1.29)	1,149 (775-1,792)
Bronsteen *et al.*, 2013, [32]	74/182,554	0.41 (0.32-0.51)	2,469 (1,965-3,145)
Hagesawa *et al.*, 2015, [33]	21/8,176	2.57 (1.59-3.92)	389 (255-629)
Catanzarite *et al.*, 2016, [10]	96/100,481	0.96 (0.77-1.17)	1,047 (857-1,292)
Kulkarni *et al.*, 2017, [34]	35/56,000	0.63 (0.44-0.87)	1,600 (1,151-2,299)
Nohuz *et al.*, 2017, [35]	8/18,152	0.44 (0.19-0.87)	2,268 (1,152-5,263)
Sullivan *et al.*, 2017, [11]	63/294,045	0.21 (0.17-0.27)	4,673 (3,650-6,061)
Yeaton-Massey *et al.*, 2019, [36]	586/945,950	0.62 (0.57-0.67)	1,616 (1,488-1,754)
Derisbourg *et al.*, 2019, [37]	4/1,620	2.47 (0.67-6.31)	405 (158-1,486)
Klahr *et al.*, 2019, [38]	61/37,236	1.64 (1.25-2.10)	610 (476-800)
La *et al.*, 2020, [39]	19/56,045	0.34 (0.20-0.53)	2,950 (1,890-4,902)
Zhang *et al.*, 2020, [12]	21/26,830	0.78 (0.49-1.20)	1,277 (836-2,062)
Gross *et al.*, 2021, [40]	21/5,905	3.56 (2.20-5.43)	281 (184-455)
Sutera *et al.*, 2021, [41]	24/89,600	0.27 (0.17-0.40)	3,731 (2,506-5,814)
Liu *et al.*, 2021, [42]	157/79,647	1.97 (1.68-2.30)	508 (435-595)
Kamijo *et al.*, 2022, [43]	14/8,723	1.60 (0.88-2.69)	625 (372-1,139)
**Pooled analysis**	**1,320/2,278,561**	**0.79 (0.59-1.01)**	**1,271 (990-1,692)**
Cochrane Q (DF)	550.11 (23)		
I^2 (inconsistency) (%) (95%CI)	95.8 (94.7-96.7)		
Egger bias (p value)	2.1799 (0.1692)		

For peer review only

For peer review only - http://bmjopen.bmj.com/site/about/guidelines.xhtml

Table 2. Results from meta-analysis using a random effects model to estimate summary statistics for incidence (95% confidence interval) of vasa praevia (VP) based on screening cohort studies that reported a specific policy of screening for VP during the study period.

Author (year)	n/N	n/1,000 pregnancies	1 per *n* pregnancies
Baulies *et al.*, 2007, [26]	9/12,063	0.75 (0.34-1.42)	1,340 (706-2,933)
Rebarber *et al.*, 2013, [31]	24/27,573	0.87 (0.56-1.29)	1,149 (775-1,792)
Catanzarite *et al.*, 2016, [10]	96/100,481	0.96 (0.77-1.17)	1,047 (857-1,292)
Kulkarni *et al.*, 2017, [34]	35/56,000	0.63 (0.44-0.87)	1,600 (1,151-2,299)
Nohuz *et al.*, 2017, [35]	8/18,152	0.44 (0.19-0.87)	2,268 (1,152-5,263)
Sullivan *et al.*, 2017, [11]	63/294,045	0.21 (0.17-0.27)	4,673 (3,650-6,061)
Klahr *et al.*, 2019, [38]	61/37,236	1.64 (1.25-2.10)	610 (476-800)
La *et al.*, 2020, [39]	19/56,045	0.34 (0.20-0.53)	2,950 (1,890-4,902)
Zhang *et al.*, 2020, [12]	21/26,830	0.78 (0.49-1.20)	1,277 (836-2,062)
Gross *et al.*, 2021, [40]	21/5,905	3.56 (2.20-5.43)	281 (184-455)
Sutera *et al.*, 2021, [41]	24/89,600	0.27 (0.17-0.40)	3,731 (2,506-5,814)
Kamijo *et al.*, 2022, [43]	14/8,723	1.60 (0.88-2.69)	625 (372-1,139)
**Pooled analysis**	**395/732,654**	**0.82 (0.53-1.18)**	**1,218 (847-1,901)**
Cochrane Q (DF)	222.18 (11)		
I^2 (inconsistency) (%) (95%CI)	95.1 (92.9-96.5)		
Egger bias (p value)	6.1508 (0.0092)		

For peer review only - http://bmjopen.bmj.com/site/about/guidelines.xhtml

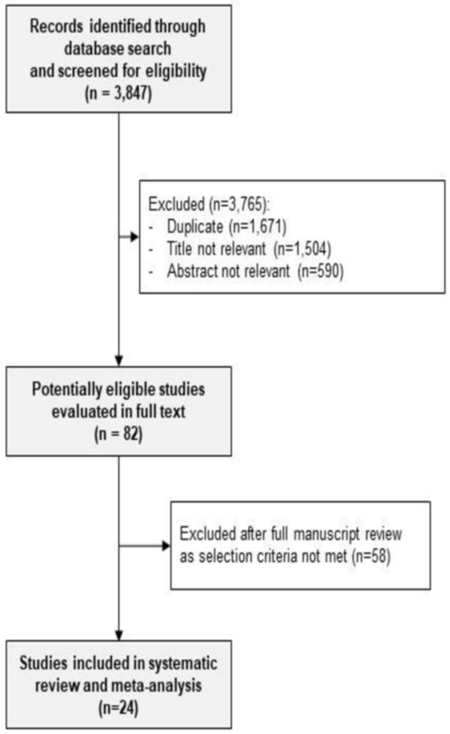

Figure 1. Flow chart demonstrating process of selection of studies included in the systematic review

89x89mm (300 x 300 DPI)

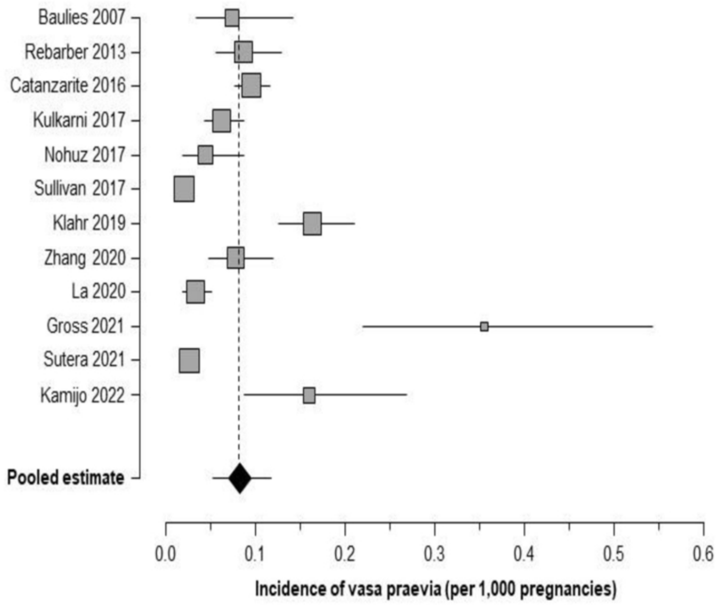

Figure 2. Forest plot demonstrating incidence of vasa praevia in studies reporting results of routine screening in low-risk unselected pregnancies. Incidence (%; 95% confidence intervals) is reported as number of cases per 1,000 pregnancies.

89x89mm (300 x 300 DPI)

Supplementary table S1: Search Strategy for study

**MEDLINE**
1. Medline(Vasa praevia OR vasa previa).ti,ab<br>2. Medline(abnormal cord insertion).ti,ab<br>3. Medline(marginal cord insertion).ti,ab<br>4. Medline(Velamentous cord insertion).ti,ab<br>5. Medline(bilobed placenta).ti,ab<br>6. Medline(succenturiate lobe).ti,ab<br>7. Medline(accessory lobe).ti,ab<br>8. Medline*(1 OR 2 OR 3 OR 4 OR 5 OR 6 OR 7)*
**CINAHL**
1. CINAHL(Vasa praevia OR vasa previa).ti,ab<br>2. CINAHL(abnormal cord insertion).ti,ab<br>3. CINAHL(marginal cord insertion).ti,ab<br>4. CINAHL(Velamentous cord insertion).ti,ab<br>5. CINAHL(bilobed placenta).ti,ab<br>6. CINAHL(succenturiate lobe).ti,ab<br>7. CINAHL(accessory lobe).ti,ab<br>8. CINAHL*(1 OR 2 OR 3 OR 4 OR 5 OR 6 OR 7)*
**PubMed**
1. PubMed(Vasa praevia OR vasa previa).ti,ab<br>2. PubMed(abnormal cord insertion).ti,ab<br>3. PubMed(marginal cord insertion).ti,ab<br>4. PubMed(Velamentous cord insertion).ti,ab<br>5. PubMed(bilobed placenta).ti,ab<br>6. PubMed(succenturiate lobe).ti,ab<br>7. PubMed(accessory lobe).ti,ab<br>8. PubMed*(1 OR 2 OR 3 OR 4 OR 5 OR 6 OR 7)*
**EMBASE**
1. EMBASE(Vasa praevia OR vasa previa).ti,ab<br>2. EMBASE(abnormal cord insertion).ti,ab<br>3. EMBASE(marginal cord insertion).ti,ab<br>4. EMBASE(Velamentous cord insertion).ti,ab<br>5. EMBASE(bilobed placenta).ti,ab<br>6. EMBASE(succenturiate lobe).ti,ab<br>7. EMBASE(accessory lobe).ti,ab<br>8. EMBASE*(1 OR 2 OR 3 OR 4 OR 5 OR 6 OR 7)*

For peer review only

Supplementary Table 4. Characteristics of the included studies

Author (year)	Study design	Study location	Population	Study period	VP Cases	Population screened	Characteristics and outcomes	Postnatal confirmation of VP
Baulies *et al.*, 2007[26]	Retrospective cohort study	Spain	Unselected pregnancies	2000-2005	9	12,063	Risk factors, delivery and neonatal outcomes	Yes
Rebarber *et al.*, 2013[31]	Retrospective cohort study	United States	Unselected pregnancies	2005-2012	24	27,573	Resolution rate, delivery outcomes, hospital stay	Yes
Catanzarite *et al.*, 2016[10]	Retrospective cohort study	United States	Unselected pregnancies	2003-2015	96	100,481	Risk factors, Antepartum haemorrhage, tocolysis, delivery and neonatal outcomes	Yes
Kulkarni *et al.*, 2017[34]	Retrospective cohort study	United States	Unselected pregnancies	2009-2017	35	56,000	Risk factors, Antepartum haemorrhage, delivery and neonatal outcomes	Yes
Nohuz *et al.*, 2017[35]	Retrospective cohort study	France	Unselected pregnancies	2011-2015	8	18,152	Risk factors, Antepartum haemorrhage, delivery and neonatal outcomes	Yes
Sullivan *et al.*, 2017[11]	Population cohort study	Australia	Unselected pregnancies	2013-2014	63	294,045	Risk factors, Antepartum haemorrhage, delivery and	Yes

For peer review only

							neonatal outcomes	
Klahr *et al.*, 2019[38]	Retrospective cohort study	United States	Unselected pregnancies	2005-2018	61	37,236	Maternal characteristics, placenta and vessel types	Yes
La *et al.*, 2020[39]	Retrospective cohort study	Australia	Unselected pregnancies	2007-2017	19	56,045	Maternal characteristics, Antepartum haemorrhage, Preterm ROM, delivery and neonatal outcomes	Yes
Zhang *et al.*, 2020[12]	Retrospective cohort study	United Kingdom	Unselected pregnancies	2011-2018	21	26,830	Maternal characteristics, delivery and neonatal outcomes	Yes
Gross *et al.*, 2021[40]	Retrospective cohort study	Austria	Unselected pregnancies	2018-2019	21	5,905	Maternal characteristics, delivery and neonatal outcomes	Yes
Sutera *et al.*, 2021[41]	Retrospective cohort study	Italy	Unselected pregnancies	2007-2018	24	89,600	Maternal characteristics, delivery and neonatal outcomes	Yes
Kamijo *et al.*, 2022[43]	Retrospective cohort study	Japan	Unselected pregnancies	2010-2020	14	8,723	Type of VP, gestation at delivery, Birth weight, neonatal Hb	Yes

VP = Vasa praevia; ROM = rupture of membranes

For peer review only

Supplementary table 2. Methodological assessment of included studies based on Newcastle – Ottawa Score Summary

Study	Selection	Comparability	Outcome	NOS Score
Lee *et al.*, 2000[24]	★★★	★	★★★	7
Catanzarite *et al.*, 2001[8]	★★★	★	★★★	7
Schachter *et al.*, 2002[25]	★★★	★	★★★	7
Baulies *et al.*, 2007[26]	★★★	★	★★★	7
Suzuki *et al.*, 2008[27]	★★★	★	★★★	7
Smorgick *et al.*, 2010[28]	★★★	-	★★★	6
Hasegawa *et al.*, 2010[29]	★★★	★★	★★★	8
Kanda *et al.*, 2011[30]	★★★	★	★★★	7
Rebarber *et al.*, 2013[31]	★★★	★	★★★	7
Bronsteen *et al.*, 2013[32]	★★★	★	★★★	7
Hasegawa *et al.*, 2015[33]	★★★	★	★★★	7
Catanzarite *et al.*, 2016[10]	★★★	★	★★★	7
Kulkarni *et al.*, 2017[34]	★★★	★	★★★	7
Nohuz *et al.*, 2017[35]	★★★	★	★★★	7
Sullivan *et al.*, 2017[11]	★★★	★	★★★	7
Yeaton-Massey *et al.*, 2019[36]	★★	★	★★★	6
Derisbourg *et al.*, 2019[37]	★★★	★	★★★	7
Klahr *et al.*, 2019[38]	★★	★	★★★	6
La *et al.*, 2020[39]	★★★	★	★★★	7
Zhang *et al.*, 2020[12]	★★★	★	★★★	7
Gross *et al.*, 2021[40]	★★★	★	★★★	7
Sutera *et al.*, 2021[41]	★★★	★	★★★	7
Liu *et al.*, 2021[42]	★★★	★	★★★	7
Kamijo *et al.*, 2022[43]	★★★	★	★★★	7

For peer review only

Supplementary table S3. The Preferred Reporting Items for Systematic Reviews and Meta-analysis: The PRISMA checklist 2020

Section and Topic	Item #	Checklist item	Location where item is reported
**TITLE**			
Title	1	Identify the report as a systematic review.	1
**ABSTRACT**			
Abstract	2	See the PRISMA 2020 for Abstracts checklist.	2
**INTRODUCTION**			
Rationale	3	Describe the rationale for the review in the context of existing knowledge.	4
Objectives	4	Provide an explicit statement of the objective(s) or question(s) the review addresses.	4
**METHODS**			
Eligibility criteria	5	Specify the inclusion and exclusion criteria for the review and how studies were grouped for the syntheses.	5
Information sources	6	Specify all databases, registers, websites, organisations, reference lists and other sources searched or consulted to identify studies. Specify the date when each source was last searched or consulted.	5
Search strategy	7	Present the full search strategies for all databases, registers and websites, including any filters and limits used.	5 (Supplementary table 1)
Selection process	8	Specify the methods used to decide whether a study met the inclusion criteria of the review, including how many reviewers screened each record and each report retrieved, whether they worked independently, and if applicable, details of automation tools used in the process.	5
Data collection process	9	Specify the methods used to collect data from reports, including how many reviewers collected data from each report, whether they worked independently, any processes for obtaining or confirming data from study investigators, and if applicable, details of automation tools used in the process.	5
Data items	10a	List and define all outcomes for which data were sought. Specify whether all results that were compatible with each outcome domain in each study were sought (e.g. for all measures, time points, analyses), and if not, the methods used to decide which results to collect.	5
	10b	List and define all other variables for which data were sought (e.g. participant and intervention characteristics, funding sources). Describe any assumptions made about any missing or unclear information.	5,6
Study risk of bias assessment	11	Specify the methods used to assess risk of bias in the included studies, including details of the tool(s) used, how many reviewers assessed each study and whether they worked independently, and if applicable, details of automation tools used in the process.	5,6
Effect measures	12	Specify for each outcome the effect measure(s) (e.g. risk ratio, mean difference) used in the synthesis or presentation of results.	6
Synthesis	13a	Describe the processes used to decide which studies were eligible for each synthesis (e.g. tabulating the study	5

For peer review only

Section and Topic	Item #	Checklist item	Location where item is reported
methods		intervention characteristics and comparing against the planned groups for each synthesis (item #5)).	
	13b	Describe any methods required to prepare the data for presentation or synthesis, such as handling of missing summary statistics, or data conversions.	6
	13c	Describe any methods used to tabulate or visually display results of individual studies and syntheses.	6
	13d	Describe any methods used to synthesize results and provide a rationale for the choice(s). If meta-analysis was performed, describe the model(s), method(s) to identify the presence and extent of statistical heterogeneity, and software package(s) used.	6
	13e	Describe any methods used to explore possible causes of heterogeneity among study results (e.g. subgroup analysis, meta-regression).	6
	13f	Describe any sensitivity analyses conducted to assess robustness of the synthesized results.	NA
Reporting bias assessment	14	Describe any methods used to assess risk of bias due to missing results in a synthesis (arising from reporting biases).	6
Certainty assessment	15	Describe any methods used to assess certainty (or confidence) in the body of evidence for an outcome.	NA
**RESULTS**			
Study selection	16a	Describe the results of the search and selection process, from the number of records identified in the search to the number of studies included in the review, ideally using a flow diagram.	6<br>Figure 1
	16b	Cite studies that might appear to meet the inclusion criteria, but which were excluded, and explain why they were excluded.	6
Study characteristics	17	Cite each included study and present its characteristics.	6,7
Risk of bias in studies	18	Present assessments of risk of bias for each included study.	7<br>Supplementary table 2
Results of individual studies	19	For all outcomes, present, for each study: (a) summary statistics for each group (where appropriate) and (b) an effect estimate and its precision (e.g. confidence/credible interval), ideally using structured tables or plots.	7,8; Table 1, Figure 2
Results of syntheses	20a	For each synthesis, briefly summarise the characteristics and risk of bias among contributing studies.	7; Supplementary table 2
	20b	Present results of all statistical syntheses conducted. If meta-analysis was done, present for each the summary estimate and its precision (e.g. confidence/credible interval) and measures of statistical heterogeneity. If comparing groups, describe the direction of the effect.	7,8; Table 1, Figure 2
	20c	Present results of all investigations of possible causes of heterogeneity among study results.	7; Table 1

For peer review only

Section and Topic	Item #	Checklist item	Location where item is reported
	20d	Present results of all sensitivity analyses conducted to assess the robustness of the synthesized results.	NA
Reporting biases	21	Present assessments of risk of bias due to missing results (arising from reporting biases) for each synthesis assessed.	NA
Certainty of evidence	22	Present assessments of certainty (or confidence) in the body of evidence for each outcome assessed.	NA
**DISCUSSION**			
Discussion	23a	Provide a general interpretation of the results in the context of other evidence.	8,9
	23b	Discuss any limitations of the evidence included in the review.	9,10
	23c	Discuss any limitations of the review processes used.	10
	23d	Discuss implications of the results for practice, policy, and future research.	9
**OTHER INFORMATION**			
Registration and protocol	24a	Provide registration information for the review, including register name and registration number, or state that the review was not registered.	5
	24b	Indicate where the review protocol can be accessed, or state that a protocol was not prepared.	5, PROSPERO website
	24c	Describe and explain any amendments to information provided at registration or in the protocol.	NA
Support	25	Describe sources of financial or non-financial support for the review, and the role of the funders or sponsors in the review.	6,11
Competing interests	26	Declare any competing interests of review authors.	11
Availability of data, code and other materials	27	Report which of the following are publicly available and where they can be found: template data collection forms; data extracted from included studies; data used for all analyses; analytic code; any other materials used in the review.	On request from authors

For peer review only

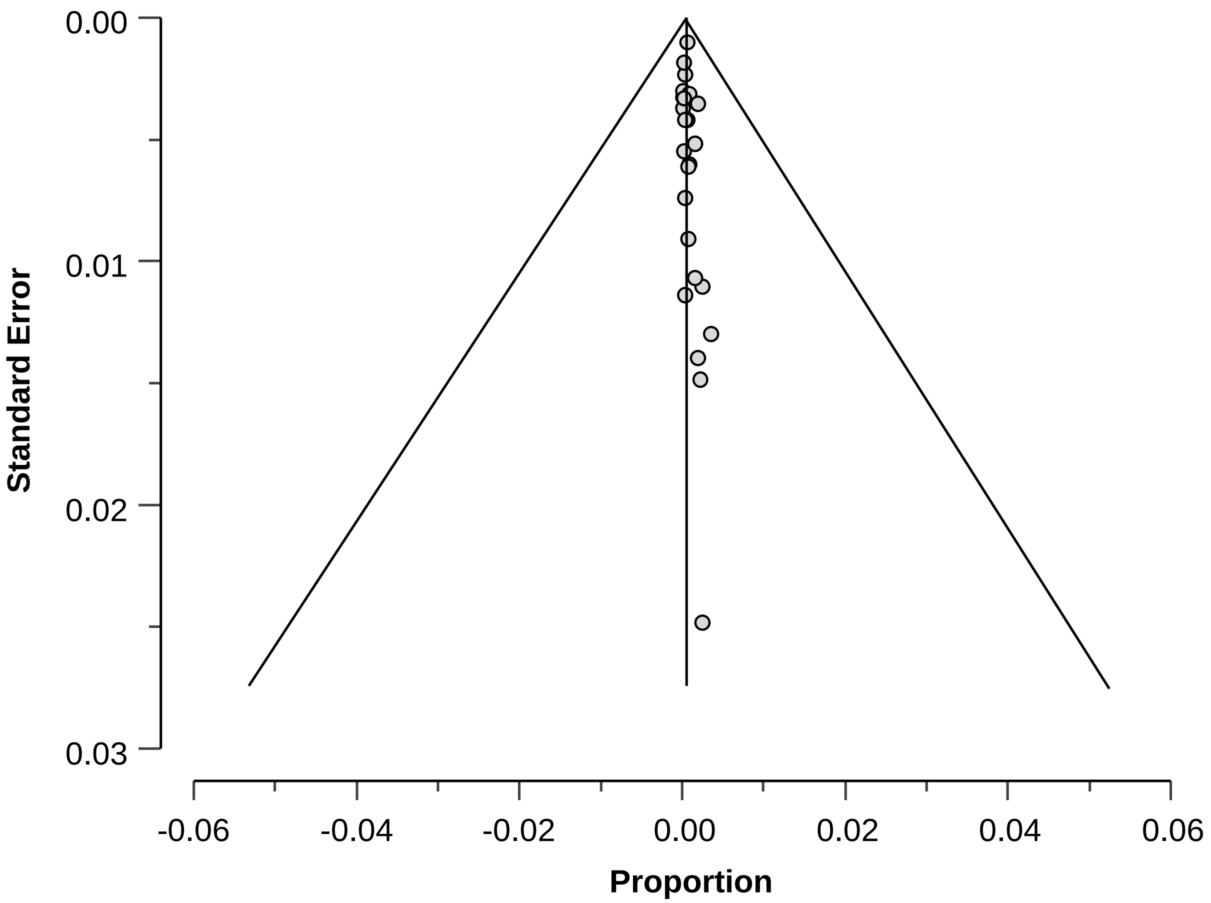

Supplementary Figure F1.

Supplementary table S3. The Preferred Reporting Items for Systematic Reviews and Meta-analysis: The PRISMA checklist 2020

Section and Topic	Item #	Checklist item	Location where item is reported
**TITLE**			
Title	1	Identify the report as a systematic review.	1
**ABSTRACT**			
Abstract	2	See the PRISMA 2020 for Abstracts checklist.	2
**INTRODUCTION**			
Rationale	3	Describe the rationale for the review in the context of existing knowledge.	4
Objectives	4	Provide an explicit statement of the objective(s) or question(s) the review addresses.	4
**METHODS**			
Eligibility criteria	5	Specify the inclusion and exclusion criteria for the review and how studies were grouped for the syntheses.	5
Information sources	6	Specify all databases, registers, websites, organisations, reference lists and other sources searched or consulted to identify studies. Specify the date when each source was last searched or consulted.	5
Search strategy	7	Present the full search strategies for all databases, registers and websites, including any filters and limits used.	5 (Supplementary table 1)
Selection process	8	Specify the methods used to decide whether a study met the inclusion criteria of the review, including how many reviewers screened each record and each report retrieved, whether they worked independently, and if applicable, details of automation tools used in the process.	5
Data collection process	9	Specify the methods used to collect data from reports, including how many reviewers collected data from each report, whether they worked independently, any processes for obtaining or confirming data from study investigators, and if applicable, details of automation tools used in the process.	5
Data items	10a	List and define all outcomes for which data were sought. Specify whether all results that were compatible with each outcome domain in each study were sought (e.g. for all measures, time points, analyses), and if not, the methods used to decide which results to collect.	5
	10b	List and define all other variables for which data were sought (e.g. participant and intervention characteristics, funding sources). Describe any assumptions made about any missing or unclear information.	5,6
Study risk of bias assessment	11	Specify the methods used to assess risk of bias in the included studies, including details of the tool(s) used, how many reviewers assessed each study and whether they worked independently, and if applicable, details of automation tools used in the process.	5,6
Effect measures	12	Specify for each outcome the effect measure(s) (e.g. risk ratio, mean difference) used in the synthesis or presentation of results.	6
Synthesis	13a	Describe the processes used to decide which studies were eligible for each synthesis (e.g. tabulating the study	5

For peer review only

Section and Topic	Item #	Checklist item	Location where item is reported
methods		intervention characteristics and comparing against the planned groups for each synthesis (item #5)).	
	13b	Describe any methods required to prepare the data for presentation or synthesis, such as handling of missing summary statistics, or data conversions.	6
	13c	Describe any methods used to tabulate or visually display results of individual studies and syntheses.	6
	13d	Describe any methods used to synthesize results and provide a rationale for the choice(s). If meta-analysis was performed, describe the model(s), method(s) to identify the presence and extent of statistical heterogeneity, and software package(s) used.	6
	13e	Describe any methods used to explore possible causes of heterogeneity among study results (e.g. subgroup analysis, meta-regression).	6
	13f	Describe any sensitivity analyses conducted to assess robustness of the synthesized results.	NA
Reporting bias assessment	14	Describe any methods used to assess risk of bias due to missing results in a synthesis (arising from reporting biases).	6
Certainty assessment	15	Describe any methods used to assess certainty (or confidence) in the body of evidence for an outcome.	NA
**RESULTS**			
Study selection	16a	Describe the results of the search and selection process, from the number of records identified in the search to the number of studies included in the review, ideally using a flow diagram.	6<br>Figure 1
	16b	Cite studies that might appear to meet the inclusion criteria, but which were excluded, and explain why they were excluded.	6
Study characteristics	17	Cite each included study and present its characteristics.	6,7
Risk of bias in studies	18	Present assessments of risk of bias for each included study.	7<br>Supplementary table 2
Results of individual studies	19	For all outcomes, present, for each study: (a) summary statistics for each group (where appropriate) and (b) an effect estimate and its precision (e.g. confidence/credible interval), ideally using structured tables or plots.	7,8; Table 1, Figure 2
Results of syntheses	20a	For each synthesis, briefly summarise the characteristics and risk of bias among contributing studies.	7; Supplementary table 2
	20b	Present results of all statistical syntheses conducted. If meta-analysis was done, present for each the summary estimate and its precision (e.g. confidence/credible interval) and measures of statistical heterogeneity. If comparing groups, describe the direction of the effect.	7,8; Table 1, Figure 2
	20c	Present results of all investigations of possible causes of heterogeneity among study results.	7; Table 1

For peer review only

Section and Topic	Item #	Checklist item	Location where item is reported
	20d	Present results of all sensitivity analyses conducted to assess the robustness of the synthesized results.	NA
Reporting biases	21	Present assessments of risk of bias due to missing results (arising from reporting biases) for each synthesis assessed.	NA
Certainty of evidence	22	Present assessments of certainty (or confidence) in the body of evidence for each outcome assessed.	NA
**DISCUSSION**			
Discussion	23a	Provide a general interpretation of the results in the context of other evidence.	8,9
	23b	Discuss any limitations of the evidence included in the review.	9,10
	23c	Discuss any limitations of the review processes used.	10
	23d	Discuss implications of the results for practice, policy, and future research.	9
**OTHER INFORMATION**			
Registration and protocol	24a	Provide registration information for the review, including register name and registration number, or state that the review was not registered.	5
	24b	Indicate where the review protocol can be accessed, or state that a protocol was not prepared.	5, PROSPERO website
	24c	Describe and explain any amendments to information provided at registration or in the protocol.	NA
Support	25	Describe sources of financial or non-financial support for the review, and the role of the funders or sponsors in the review.	6,11
Competing interests	26	Declare any competing interests of review authors.	11
Availability of data, code and other materials	27	Report which of the following are publicly available and where they can be found: template data collection forms; data extracted from included studies; data used for all analyses; analytic code; any other materials used in the review.	On request from authors

For peer review only
